# Convolutional, State-Space and Transformer Liver-Tumor Segmentation Models Trained on Hepatocellular Carcinoma Fail on the Same External Cases: Lesion Size Outweighs Architecture in Cross-Dataset Generalization

**DOI:** 10.3390/cancers18203362

**Published:** 2026-10-17

**Authors:** Hüseyin Kutlu, Cemil Çolak, Sami Akbulut

**Affiliations:** 1 Department of Biostatistics and Medical Informatics, Faculty of Medicine, Adıyaman University, 02040 Adıyaman, Türkiye; hkutlu@adiyaman.edu.tr; 2 Department of Biostatistics and Medical Informatics, Faculty of Medicine, İnönü University, 44280 Malatya, Türkiye; akbulutsami@gmail.com; 3 Department of Surgery and Liver Transplantation, Faculty of Medicine, İnönü University, 44280 Malatya, Türkiye

**Keywords:** hepatocellular carcinoma, liver-tumor segmentation, computed tomography, lesion size, deep learning, external validation, domain shift, model calibration, selective prediction

## Abstract

Background/Objectives: Architectural innovation is widely assumed to improve the cross-institutional generalization of tumor segmentation models, yet this is rarely tested. We compared three architectural families under an identical, leak-safe protocol in hepatocellular carcinoma (HCC) segmentation. Methods: Convolutional (nnU-Net ResEnc-M), state-space (U-Mamba Bot) and transformer (SwinUNETR) models were trained on HCC-TACE-Seg (*n* = 103) with identical patient-disjoint five-fold splits, then applied without adaptation to 3D-IRCADb (*n* = 15) and LiTS (*n* = 118). We assessed discrimination, case-level failure concordance, lesion-volume dependence, calibration and selective prediction. Results: Internal performance differed (Dice 0.685, 0.662, 0.479; Friedman *p* = 0.022) but external performance was low and largely convergent: the convolutional and state-space models were formally equivalent on both external cohorts (pre-specified margin ±0.05 Dice), whereas the transformer was inferior on LiTS (0.222; *p* < 0.0001) and inconclusive on 3D-IRCADb. Case-level Dice was highly concordant across all pairings (Spearman ρ = 0.84–0.96; 0.67–0.81 on LiTS after adjustment for lesion volume), the same cases failing across architectures. Lesion volume predicted performance in all six architecture × cohort combinations; lesions < 5 mL failed completely in 61–76% of cases and formed 1.9% of the development cohort against 31.4% of LiTS. Neither liver-mask constraint nor ensembling recovered performance. Deferring the 30% least-confident LiTS cases raised Dice to 0.516 (0.450 when only empty predictions were removed). Conclusions: Under a single-source, TACE-treated HCC training regime, cross-dataset failure of liver-tumor segmentation was governed primarily by lesion size rather than by architectural family; confidence-based selective prediction offers a deployable mitigation.

## Simple Summary

Artificial intelligence models can outline liver tumors on CT scans, but a model trained at one hospital often performs poorly on scans from another. The common response has been to design more sophisticated model architectures. We tested whether this works. Using three fundamentally different model types—convolutional, state-space, and transformer—trained identically on the same 103 patients, we measured how each performed on 133 patients from two unrelated hospitals. Although the models differed considerably on their own data, two of the three architectures performed equivalently on outside data, and all three failed on the same patients. These findings apply to models trained on a single, homogeneous cohort of treated hepatocellular carcinoma and tested on mixed-etiology liver-tumor datasets. What determined success was not the model but the tumor: small tumors were missed in roughly two of three cases, whereas large tumors were segmented reliably. The training scans contained almost no small tumors, while a third of the outside scans did. All models were poorly calibrated, yet their confidence still tracked how accurate they actually were, meaning unreliable cases can be flagged automatically for radiologist review. These findings suggest that efforts to improve cross-hospital reliability should focus on data diversity, small-lesion sensitivity, and safety mechanisms rather than on new architectures alone.

## 1. Introduction

Hepatocellular carcinoma (HCC) ranks as the third leading cause of cancer-related mortality worldwide [1], against a background of some two million annual deaths from liver disease [2], with incidence patterns shifting geographically and by etiology over time [3]. Quantitative assessment of tumor burden on contrast-enhanced CT underpins staging, treatment selection, and response evaluation, and increasingly feeds downstream models for surgical planning and recurrence prediction that depend on reliable tumor delineation [4,5]. Manual delineation is time-consuming and subject to substantial inter-reader variability, which has motivated a large body of work on automated tumor segmentation.

Progress in this field is conventionally measured by architectural innovation. The convolutional encoder–decoder established the modern baseline, with self-configuring pipelines making strong convolutional models widely accessible [6]. Vision transformers were subsequently introduced on the argument that self-attention captures long-range context that convolutions cannot [7,8], and, more recently, selective state-space models [9] have been proposed as a way to obtain global receptive fields at linear rather than quadratic cost [10,11,12]. Each generation has been reported to improve segmentation accuracy on held-out data from the same source as the training set.

Two observations motivate the present study. First, the great majority of these comparisons are internal: the models are ranked on cross-validation folds drawn from a single cohort or benchmark partition [13,14], which measures fit to that cohort’s acquisition protocol, patient mix, and annotation convention rather than transferability. A systematic review of 22 HCC prediction studies found external validation cohorts in only two, with a mean Radiomics Quality Score of 10 of a possible 36 [15]. Second, where external validation is reported it typically involves a single architecture [16,17,18]; it therefore establishes that performance degrades but not whether the degradation is a property of the model or of the task. Reported degradations are substantial and consistent across modalities: internal-to-external drops from 0.80 to 0.60 Dice in liver-tumor segmentation [16], from AUC 0.998 to 0.745 in liver cancer histopathology [19], by 8–12 accuracy points in abdominal CT classification [20], and across three external institutions in pancreatic lesion diagnosis [21]. The implicit assumption bridging these two observations—that an architecture which performs better internally will also transfer better—is rarely tested, yet it shapes how research effort is allocated.

We tested that assumption directly. Three models representing three architectural families—convolutional, state-space, and transformer—were trained on a single HCC cohort under identical splits and an identical optimization budget, and applied without adaptation to two independent external cohorts. Beyond comparing mean accuracy, we asked whether the three models fail on the same cases, what case-level property governs failure, and whether predicted probabilities remain trustworthy enough to support a clinical safety mechanism [22].

The study is deliberately framed as a test of a prevailing assumption rather than as a proposal for a new architecture. Its contribution is diagnostic: it identifies where the cross-dataset problem does not lie, quantifies where it does, and evaluates one mitigation that is available without retraining.

## 2. Materials and Methods

### 2.1. Study Design

We conducted a retrospective, fully reproducible benchmarking study using three publicly available CT datasets. A single development cohort was used to train three segmentation models representing three distinct architectural families; both external cohorts were held out entirely and used only for inference. No external image, label, or summary statistic was used at any point during model development, hyperparameter selection, or checkpoint selection, so that the external evaluation is free of information leakage by construction.

Sample size was determined by the size of the available public cohorts rather than by a formal power calculation. The design is therefore powered to detect moderate between-architecture differences at the case level in the larger external cohort (*n* = 118) but not small differences at the fold level (*n* = 5); this constraint is quantified where relevant in the Results and revisited in the Limitations.

The study addresses four questions in sequence: (i) whether architectural family affects internal performance; (ii) whether any advantage observed internally is preserved under cross-dataset transfer; (iii) whether failures are arm-specific or case-specific, and which case-level property governs them; and (iv) whether predicted probabilities are calibrated well enough to support a confidence-based safety mechanism.

### 2.2. Datasets

- Development cohort. HCC-TACE-Seg [23], hosted by The Cancer Imaging Archive [24], comprises contrast-enhanced CT studies of patients with hepatocellular carcinoma (HCC) treated with transarterial chemoembolization (TACE), with expert tumor segmentations distributed as DICOM SEG objects. Conventional TACE deposits lipiodol within treated lesions, which alters their attenuation on follow-up CT [25]; treated HCC therefore differs radiologically from untreated disease [26]. Cases were downloaded using ‘tcia_utils’. An initial conversion with ‘pydicom_seg’ produced masks that were geometrically misaligned with their source CT volumes in a subset of cases, owing to inverted z-axis ordering of the segmentation frames. The pipeline was therefore rebuilt around ‘highdicom’, which places each segmentation frame using the SOP Instance UID of the CT slice it references, removing any reliance on inferred geometry. Where a study contained multiple CT sub-series, each was tested and the one yielding the largest non-empty tumor mask was retained. Of 105 available segmentation objects (one per patient), 2 could not be reconstructed and were excluded, giving a final development cohort of 103 patients.
- External cohort 1. 3D-IRCADb-01 [27]—contrast-enhanced abdominal CT with manual liver and lesion annotations. Only cases containing at least one liver tumor were retained (*n* = 15).
- External cohort 2. Liver-Tumor Segmentation Benchmark (LiTS), distributed as Task03 (Liver) of the Medical Segmentation Decathlon (MSD) [28,29]—multi-institutional contrast-enhanced abdominal CT assembled from seven hospitals and research institutions, containing primary and secondary tumors of varied size and lesion-to-background contrast, with liver and lesion annotations. Cases containing at least one liver tumor were retained (*n* = 118).

The two external cohorts differ from the development cohort in acquisition site, scanner, contrast phase, annotation protocol, and—critically—in treatment status: the development cohort consists exclusively of TACE-treated tumors, which exhibit lipiodol retention and post-treatment necrosis, whereas the external cohorts consist predominantly of untreated lesions. The external task is therefore best described as liver-lesion segmentation under combined domain, disease-type and annotation-protocol shift, rather than HCC segmentation in the strict sense; case-level lesion etiology is not released with either external dataset, so an HCC-only subset could not be isolated.

### 2.3. Preprocessing and Data Splits

All data were processed with the nnU-Net v2 pipeline [6]. Images were resampled to the dataset-derived target spacing of 1.245 × 0.781 × 0.781 mm and intensity-normalized using nnU-Net CT normalization (clipping to the 0.5th–99.5th foreground percentiles, followed by z-scoring with foreground mean and standard deviation). The 3D full-resolution configuration was used throughout, with a patch size of 64 × 192 × 192 and a batch size of 2.

A single patient-disjoint five-fold cross-validation split (83 training/20 validation cases per fold) was generated once and reused verbatim for all architectures (including the fold-0 sensitivity arm described in Section 2.4) via a shared ‘splits_final.json’. Fixing the splits across architectures removes split variance as a confounder in the between-architecture comparison and permits fold-level paired testing.

The segmentation target was the tumor class only; liver parenchyma was not modeled.

### 2.4. Architectures and Training

Three models were selected as representatives of the three architectural families currently dominating volumetric medical image segmentation: nnU-Net with a residual encoder [6], U-Mamba [30], and SwinUNETR [31]. Table 1 summarizes the three architectures and the training configuration used for each.

All models were trained for 250 epochs per fold, giving each architecture an identical optimization budget. Data augmentation, loss function (compound Dice + cross-entropy), sampling strategy, and patch size were identical across architectures and inherited from the nnU-Net defaults. The complete configuration of each arm, including software versions and hardware, is given in Table S3.

Two deviations from a fully identical training regime were unavoidable and are reported explicitly. First, U-Mamba required mixed-precision training to be disabled, together with a reduced learning rate and gradient-norm clipping at 12: with automatic mixed precision enabled, the Mamba selective-scan kernels produced non-finite losses from the first epoch, consistent with float16 overflow in the selective-scan recurrence. Second, SwinUNETR was trained with the reference trainer distributed with the U-Mamba repository, which uses AdamW with cosine annealing and does not employ deep supervision. We considered imposing the SGD/deep-supervision regime on SwinUNETR for strict symmetry but rejected this, since transformer segmentation networks are known to underperform substantially under SGD; training each architecture under the regime recommended by its authors yields a fairer—and, for our conclusion, a more conservative—comparison. The consequences of this choice are addressed in the Limitations. As a sensitivity analysis, a fourth arm, SwinUNETR-pretrained, was trained for fold 0 only. It is identical to the transformer arm in every respect except that the Swin encoder was initialized from the self-supervised weights of Tang et al. [32], pretrained on 5050 public CT volumes; the decoder and segmentation head were trained from scratch, and optimizer, schedule, budget and precision settings were unchanged. Because the pretraining used a different intensity normalization and patch size, the encoder is transferred rather than matched to our pipeline. This arm is compared with the from-scratch fold-0 checkpoint only (Section 3.11).

All experiments were run on a single NVIDIA RTX 6000 Ada (48 GB).

### 2.5. External Inference

For each architecture, the five fold checkpoints were applied as an ensemble using nnU-Net sliding-window inference with test-time mirroring, and softmax probability maps were retained. No fine-tuning, no domain adaptation, no intensity harmonization, and no threshold recalibration were applied to the external cohorts. Predictions were resampled to the native geometry of each external case before evaluation.

### 2.6. Evaluation Metrics

Segmentation agreement was quantified per case using the Dice similarity coefficient (DSC), Jaccard index (IoU), 95th-percentile Hausdorff distance (HD95), and average symmetric surface distance (ASSD). Predictions were resampled to the reference geometry by nearest-neighbor interpolation where the two differed. Surface distances were computed in millimeters from signed Maurer distance maps using native voxel spacing, pooling prediction-to-reference and reference-to-prediction distances, so both HD95 and ASSD are symmetric; values computed one-directionally are not directly comparable. Distance metrics are undefined when either mask is empty and were treated as missing in those cases. Cohort-level estimates are reported with nonparametric bootstrap 95% confidence intervals (2000 resamples, fixed seed). Reporting follows TRIPOD+AI [33,34] and the CLAIM 2024 update [35]; the completed checklists are provided in Tables S1 and S2 as Supplementary Material.

The generalization gap is defined as Δ = (external cohort Dice) − (internal five-fold mean Dice).

Two distinct notions of complete failure are reported, because they behave very differently across architectures and conflating them is misleading:

- Empty prediction—the model produced no tumor voxels anywhere in the volume;
- Zero overlap—the model produced tumor voxels, but with no intersection with the reference mask (DSC = 0).

A model may have zero empty predictions and still fail completely on many cases by segmenting the wrong structures.

Reference lesion volume was computed per case from the ground-truth mask using native voxel spacing and stratified as <5 mL, 5–20 mL, 20–50 mL, and >50 mL.

Lesion-wise detection was evaluated by 26-connected component analysis of the reference and predicted masks. A reference lesion was counted as detected if the union of overlapping predicted components reached IoU ≥ 0.1 with it (any-voxel overlap is also reported); a predicted component with no overlap with any reference lesion was counted as a false positive. Sensitivity was stratified by the volume of the individual lesion (<1, 1–5, 5–20, and >20 mL), which is finer than the case-level stratification because individual lesions are much smaller than total tumor burden per case.

### 2.7. Calibration and Selective Prediction

Calibration was assessed on the retained probability maps. Expected calibration error (ECE) and maximum calibration error (MCE) were computed over 15 equal-width confidence bins using voxels with predicted tumor probability >0.01, excluding the trivially confident background that would otherwise dominate the estimate. Reliability curves were derived from the same binning.

Two case-level confidence summaries were used: the mean predicted probability within the predicted tumor region, and the maximum predicted probability anywhere in the volume. The latter was examined specifically within completely failed cases to determine whether models remain confident while wrong.

For selective prediction, cases were ranked by a selection score computable without reference labels—either mean predicted confidence or predicted tumor volume—and risk–coverage behavior was evaluated by progressively deferring the lowest-ranked cases and recomputing mean DSC and complete-failure rate on the retained subset. Mean predicted confidence is undefined for cases with an empty prediction; these were assigned a confidence of zero and are therefore the first cases deferred under confidence ranking. Because this rule alone accounts for the first 19% of deferrals on LiTS, we report, alongside the confidence curve, (i) a baseline in which only empty predictions are removed and the remaining cases are retained in random order, (ii) random deferral (mean of 1000 permutations), and (iii) an oracle that defers cases in ascending order of true Dice. Risk–coverage curves were summarized by the area under the risk–coverage curve (AURC, risk = 1 − Dice, coverage 30–100%) and the excess AURC over the oracle. The lesion-volume distribution of deferred and retained cases was recorded at 70% and 50% coverage. Deferred cases correspond, operationally, to studies routed to expert review.

### 2.8. Statistical Analysis

Fold-level comparison of the three architectures used the Friedman test with post hoc paired Wilcoxon signed-rank tests (five folds). Case-level comparison in the external cohorts used the Friedman test across all cases with post hoc paired Wilcoxon tests. Case-level agreement between architectures, and associations between lesion volume, confidence, and DSC, were quantified with Spearman rank correlation. Two-sided tests were used throughout with α = 0.05; where multiple pairwise comparisons were made, Bonferroni correction was applied within each family of three pairwise comparisons (α = 0.05/3 = 0.0167); the internal fold-level, 3D-IRCADb and LiTS pairwise tests constitute three such families.

Equivalence between architectures was assessed with a two one-sided-tests procedure implemented by paired bootstrap (5000 resamples): two models were declared equivalent when the 90% confidence interval of the mean paired Dice difference lay entirely within a pre-specified margin of ±0.05 Dice, chosen as the smallest difference we would regard as practically relevant in this task.

To separate architectural concordance from the common effect of lesion size, partial Spearman correlations were computed by residualizing the rank-transformed Dice of each architecture on log10 reference volume (95% CI from 2000 case-level bootstrap resamples). As a reference for the concordance attainable between two models of the same architecture, the five fold checkpoints of each arm were applied individually to the external cohorts (single-fold inference, no test-time augmentation) and their pairwise case-level correlations were computed; cross-architecture concordance was additionally computed fold-for-fold so that ensembling does not inflate agreement.

To test whether lesion volume remained associated with performance after adjustment for co-varying cohort and acquisition properties, a linear mixed-effects model was fitted to case-level Dice with log10 reference volume, mean and standard deviation of tumor HU, log10 lesion count, slice thickness, cohort and architecture as fixed effects and a random intercept per case (statsmodels MixedLM, maximum likelihood); a volume × architecture interaction was tested separately.

Reporting adheres to TRIPOD+AI [33,34] and CLAIM 2024 [35]. All code required to reproduce the data preparation, training, inference, evaluation and figures, together with the exact five-fold split assignment and per-case results, is available at https://github.com/huseyinKutlu/hcc-crossdataset-segmentation (accessed on 1 October 2026) and permanently archived at Zenodo (doi:10.5281/zenodo.23090049). ResEnc-M was trained and applied under nnU-Net v2.8.1 (‘hccseg’ environment: Python 3.11, PyTorch 2.4.1 + CUDA 12.1); U-Mamba and SwinUNETR were trained and applied under the U-Mamba fork of nnU-Net v2.1.1 (‘umamba’ environment: Python 3.10, PyTorch 2.1.2 + CUDA 12.1, MONAI 1.3.0). All evaluation and statistical analyses were performed in Python (version 3.13.2; Python Software Foundation, Wilmington, DE, USA) using NumPy (version 2.5.1), pandas (version 2.2.3), SciPy (version 1.16.3), SimpleITK (version 2.5.3) and statsmodels (version 0.14.6). All preprocessing, splits, and evaluation code were shared.

To test whether a deferral operating point transfers across sites, confidence thresholds corresponding to fixed coverage levels were derived on 3D-IRCADb and applied unchanged to LiTS, and realized coverage and retained-subset performance were recorded.

Cohort characteristics were compared by extracting, per case, slice thickness, in-plane spacing, mean and standard deviation of Hounsfield units within the reference tumor mask, total lesion volume, and the number of connected lesion components.

As a further test of whether architectural diversity carries complementary information, we averaged the softmax tumor-probability maps of the three models—for every pairwise combination and for all three together—and evaluated the averaged maps at the same 0.5 threshold.

To quantify how much of the external deficit is attributable to anatomically implausible predictions, tumor predictions were intersected with the reference liver mask and all metrics recomputed. Liver masks were taken from the reference annotations of each external cohort (the union of liver and tumor labels for LiTS; the ‘liver’ mask series for 3D-IRCADb). Because a reference liver mask would not be available at deployment, this analysis is an upper bound corresponding to perfect liver segmentation rather than a deployable pipeline. We additionally report, per case, the fraction of predicted tumor volume falling outside the liver.

## 3. Results

Of the cases available in each source, the following were analyzed. The HCC-TACE-Seg collection yielded 105 segmentation objects, one per patient; two patients could not be reconstructed (one lacking a resolvable reference CT series and one for which no non-empty tumor mask could be recovered), leaving 103 for development. From 3D-IRCADb-01, 20 patients were retrieved and 15 contained at least one liver tumor. From MSD Task03 (Liver), 131 annotated volumes were retrieved and 118 contained at least one liver tumor. All tumor-bearing cases from both external sources were used; none were excluded for image quality or any other reason (Figure S1).

### 3.1. Internal Performance Differs Across Architectural Families

**Figure 1:**
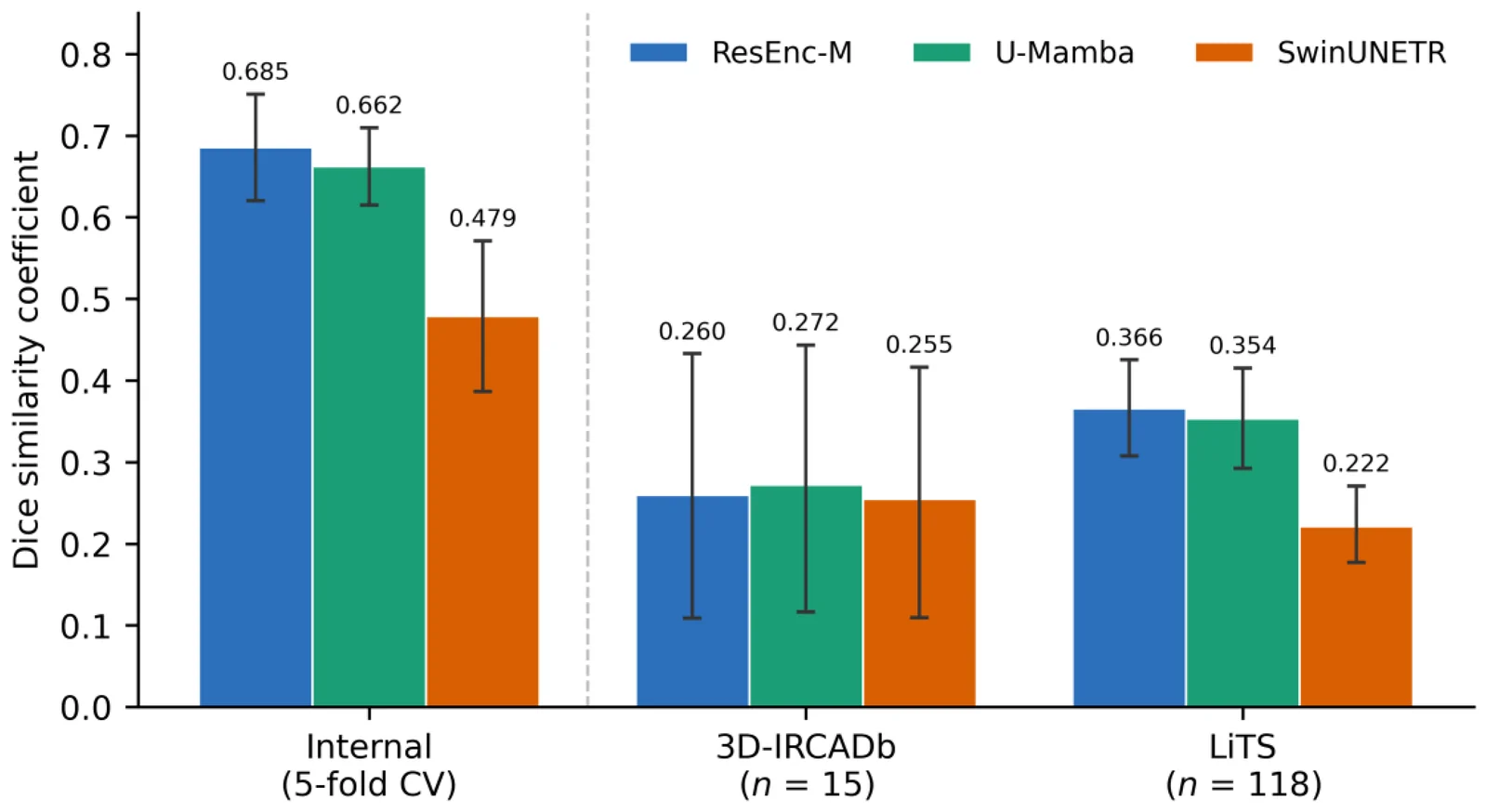
Internal and external segmentation performance for the three architectural families. The internal values are means across the five cross-validation folds with error bars showing the standard deviation across folds; the external values are cohort means with bootstrap 95% confidence intervals. The three families separate internally but converge on 3D-IRCADb, and the family with the strongest internal performance gains no external advantage.

Under identical splits and an identical epoch budget, the three architectures separated clearly on internal five-fold cross-validation (Table 2). The convolutional and state-space models performed comparably (0.685 ± 0.065 vs. 0.662 ± 0.044), whereas the transformer trailed both by roughly 0.18–0.21 DSC (0.479 ± 0.093). The Friedman test across folds was significant (χ^2^ = 7.60, *p* = 0.022). Figure 1 summarizes internal and external performance for all three architectures.

Post hoc paired tests did not reach the Bonferroni-corrected threshold (ResEnc-M vs. U-Mamba *p* = 0.313; ResEnc-M vs. SwinUNETR *p* = 0.063; U-Mamba vs. SwinUNETR *p* = 0.063). With five folds, however, 0.063 is the smallest attainable *p*-value for the Wilcoxon signed-rank test, so the transformer comparisons are at the floor of what this design can resolve: SwinUNETR ranked lowest in all five folds. The effect sizes are unambiguous—the CNN–SSM difference (0.023) lies well within the fold-to-fold standard deviations, whereas the transformer deficit (0.18–0.21) lies far outside them.

Fold 2 produced the lowest Dice for all three architectures, indicating that fold difficulty is a property of the data partition rather than of any model.

### 3.2. Internal Advantage Does Not Transfer

All three models degraded severely on both external cohorts, and the internal ordering was not preserved (Table 3).

On 3D-IRCADb the three architectures did not differ significantly (Friedman *p* = 0.627; all pairwise Wilcoxon *p* ≥ 0.374) and clustered within 0.255–0.272 despite internal Dice values spanning 0.479–0.685; on LiTS the convolutional and state-space models did not differ (0.366 vs. 0.354, *p* = 0.184), whereas the transformer was inferior (both pairwise *p* < 0.0001). Because non-significant differences do not establish equivalence, we tested equivalence formally with a pre-specified margin of ±0.05 Dice (Table 4). The convolutional and state-space models were equivalent on 3D-IRCADb (mean difference −0.012, 90% CI −0.039 to 0.017), on LiTS (0.013, −0.011 to 0.035) and in the >50 mL stratum (0.004, −0.017 to 0.025); in the smaller strata the point estimates remained below 0.02 but the confidence intervals were too wide to declare equivalence. The transformer was not equivalent to either model: on LiTS it was inferior by 0.13–0.15 Dice (both *p* < 0.0001), and this deficit was present in every volume stratum, including lesions < 5 mL (0.076, *p* = 0.011). On 3D-IRCADb (n = 15) the transformer comparisons were inconclusive—neither equivalence nor a difference could be established. The external convergence reported in this study therefore applies to the convolutional and state-space families; the transformer trailed both wherever the sample size allowed a determination.

Part of the low external performance reflects a floor: all three models scored below 0.10 Dice in 53% of 3D-IRCADb cases and 31% of LiTS cases. The convolutional–state-space equivalence is not an artifact of this floor—among the 82 LiTS cases above it, their difference remained 0.019 (*p* = 0.14)—whereas the transformer deficit widened to 0.21 when floor cases were removed, indicating that it is a property of the cases that can be segmented, not of those that cannot.

The generalization gap should not be read in isolation. SwinUNETR shows the smallest Δ on both cohorts, but only because it starts from a substantially lower internal baseline; its absolute external performance is the worst of the three. Interpreted jointly, Δ and absolute external DSC indicate that no architectural family achieved clinically usable cross-dataset performance, and that the family with the strongest internal performance gained no external advantage.

### 3.3. Failures Are Case-Specific, Not Arm-Specific

**Figure 2:**
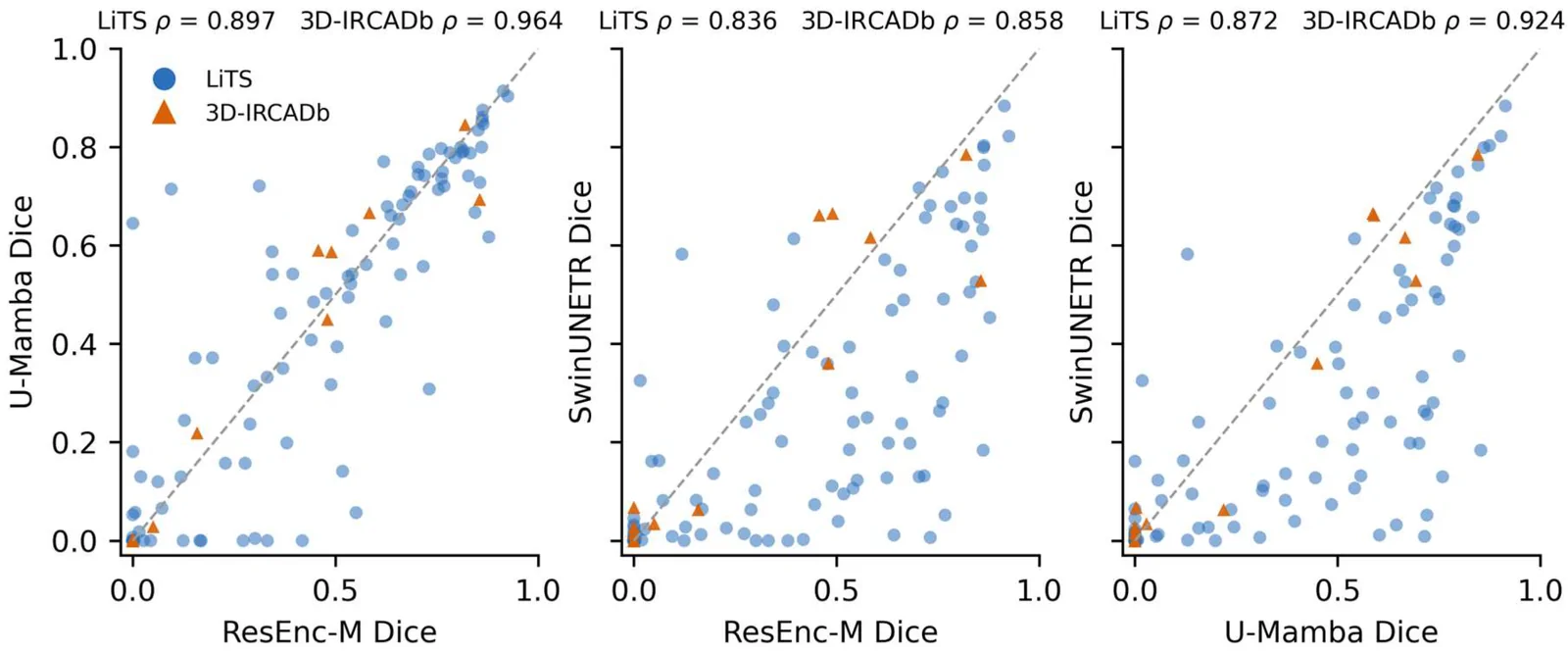
Case-level agreement between architectures on both external cohorts. Each point is one case; the dashed line is the identity. Spearman coefficients are given per cohort above each panel. Agreement is high for every pairing, including convolutional versus transformer, indicating that the same cases succeed and fail irrespective of architecture. The points clustered at the origin are cases failed completely by both models of the pair.

Case-level Dice values were highly concordant across all three architectural pairings—including the most dissimilar pairing, convolutional versus transformer (Table 5, Figure 2).

Because lesion volume affects Dice in any model, part of this concordance is expected from volume alone. Adjusting for log lesion volume reduced but did not remove it: on LiTS the partial correlations were 0.81 (CNN–SSM), 0.67 (CNN–transformer) and 0.73 (SSM–transformer), with lower confidence limits of 0.53 or above (Table 5). Roughly one fifth of the shared variance between architectures is attributable to volume; the remainder reflects case-level difficulty that is not captured by size and that all three families encounter in the same cases.

As a reference for the concordance attainable between two models of the same architecture, we applied each of the five fold checkpoints individually to the external cohorts and correlated their case-level Dice. Fold-to-fold concordance within an architecture on LiTS was 0.90 (range 0.86–0.93) for the convolutional, 0.88 (0.82–0.91) for the state-space and 0.93 (0.91–0.95) for the transformer model. Compared fold-for-fold, so that ensembling does not inflate agreement, the concordance between the convolutional and state-space models was 0.86 (0.79–0.90)—within 0.02–0.04 of the within-architecture reference—whereas transformer–convolutional (0.82) and transformer–state-space (0.85) concordance fell 0.08–0.11 below the transformer’s own fold-to-fold reference of 0.93 (Table 5). Changing the architecture between the convolutional and state-space families therefore adds little disagreement beyond that produced by resampling the training data; the transformer fails in a partly distinct set of cases, consistent with Section 3.2 and the lesion-wise detection analysis in Section 3.4.

Knowing which cases one architecture handles well predicts, with high accuracy, which cases the others handle well—despite the three models sharing no inductive bias beyond the encoder–decoder skeleton and having been trained with different optimizers. Complete failures overlapped correspondingly: largely the same cases failed across architectures. Case-level Dice, reference lesion volume and surface distances for every case and architecture are given in Table S4.

### 3.4. Lesion Volume Dominates Performance After Adjustment for Co-Varying Properties

**Figure 3:**
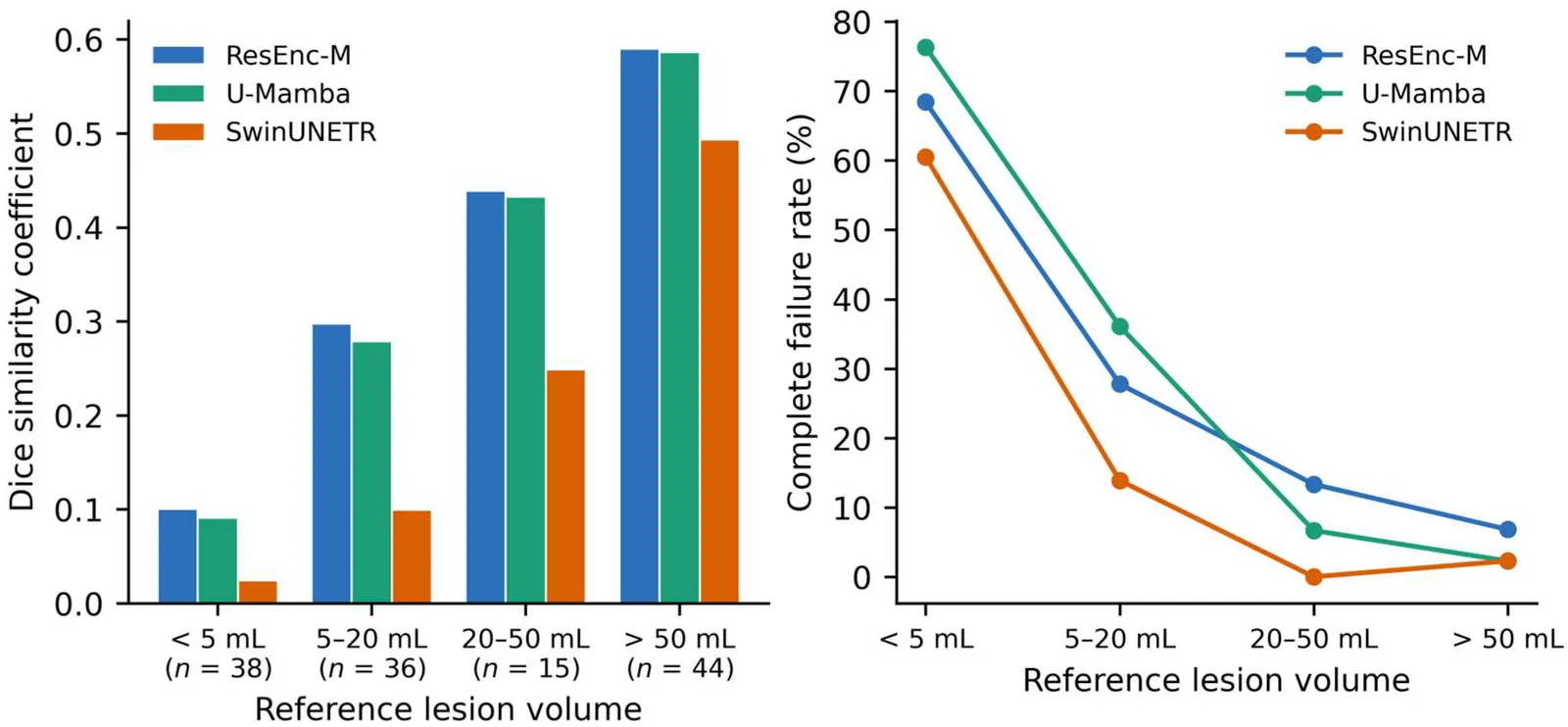
Performance stratified by reference lesion volume, both external cohorts pooled *(n* = 133). **Left**: Mean Dice per volume stratum. **Right**: Complete-failure rate, defined as zero overlap. All three architectures show the same monotone gradient. The apparently lower failure rate of the transformer in the smallest stratum should be read together with its Dice in the same stratum (0.025); it rarely abstains, but it rarely overlaps the lesion either.

Reference lesion volume was significantly associated with Dice in all six architecture × cohort combinations (Spearman ρ = 0.587–0.788, all *p* < 0.05; Table 6), and the stratified analysis shows a monotone gradient across all three architectures (Table 7, Figure 3).

Because lesion volume co-varies with attenuation, lesion count, slice thickness and cohort (Section 3.8), we fitted a mixed-effects model with all of these as covariates and a random intercept per case (Table 8). Lesion volume was the only covariate associated with Dice (0.21 per SD of log volume, *p* < 0.001); attenuation, lesion count, slice thickness and cohort contributed nothing once volume was included, and a model containing volume alone fitted better (AIC −211) than the full model (−206), whereas removing volume degraded the fit substantially (−136). The volume slope did not differ between architectures (interaction *p* ≥ 0.30), so the size dependence is architecture-invariant in slope as well as in direction. The transformer’s deficit (−0.13) persisted after adjustment, whereas the state-space model did not differ from the convolutional model (−0.01). The case random intercept accounted for 69% of the variance not explained by the fixed effects, quantifying the shared, architecture-independent case-level component identified in Section 3.3.

#### Lesion-Wise Detection

Case-level Dice on total tumor burden can be dominated by the largest lesion in multifocal cases, so we additionally evaluated detection per individual reference lesion (Table 9). LiTS contained 908 individual lesions, 565 of them (62%) below 1 mL. Lesion-wise sensitivity showed the same monotone gradient as case-level Dice for all three architectures: for ResEnc-M, 3% of sub-milliliter lesions were detected, rising to 27% at 1–5 mL, 59% at 5–20 mL and 81% above 20 mL; U-Mamba was within four percentage points at every stratum. Overall lesion-wise sensitivity (0.20) was roughly half the 0.46–0.55 lesion-wise recall reported for in-domain LiTS submissions [28], consistent with the case-level ceiling analysis in Section 4.5. The transformer showed a distinct profile: its any-overlap sensitivity was the highest of the three (0.46 overall; 0.97 above 20 mL), but its IoU-based sensitivity was the lowest (0.14; 0.60 above 20 mL), and it produced a mean of 37 false-positive components per LiTS case (median 16.5) against 0.7–1.0 for the other two models. The transformer thus reaches lesions more often than the other architectures but with fragmented, spatially diffuse predictions and a large number of spurious components—the lesion-level counterpart of the extra-hepatic leakage reported in Section 3.9.

### 3.5. Miscalibration Is Cohort-Dependent, and Failure Confidence Is Arm-Dependent

**Figure 4:**
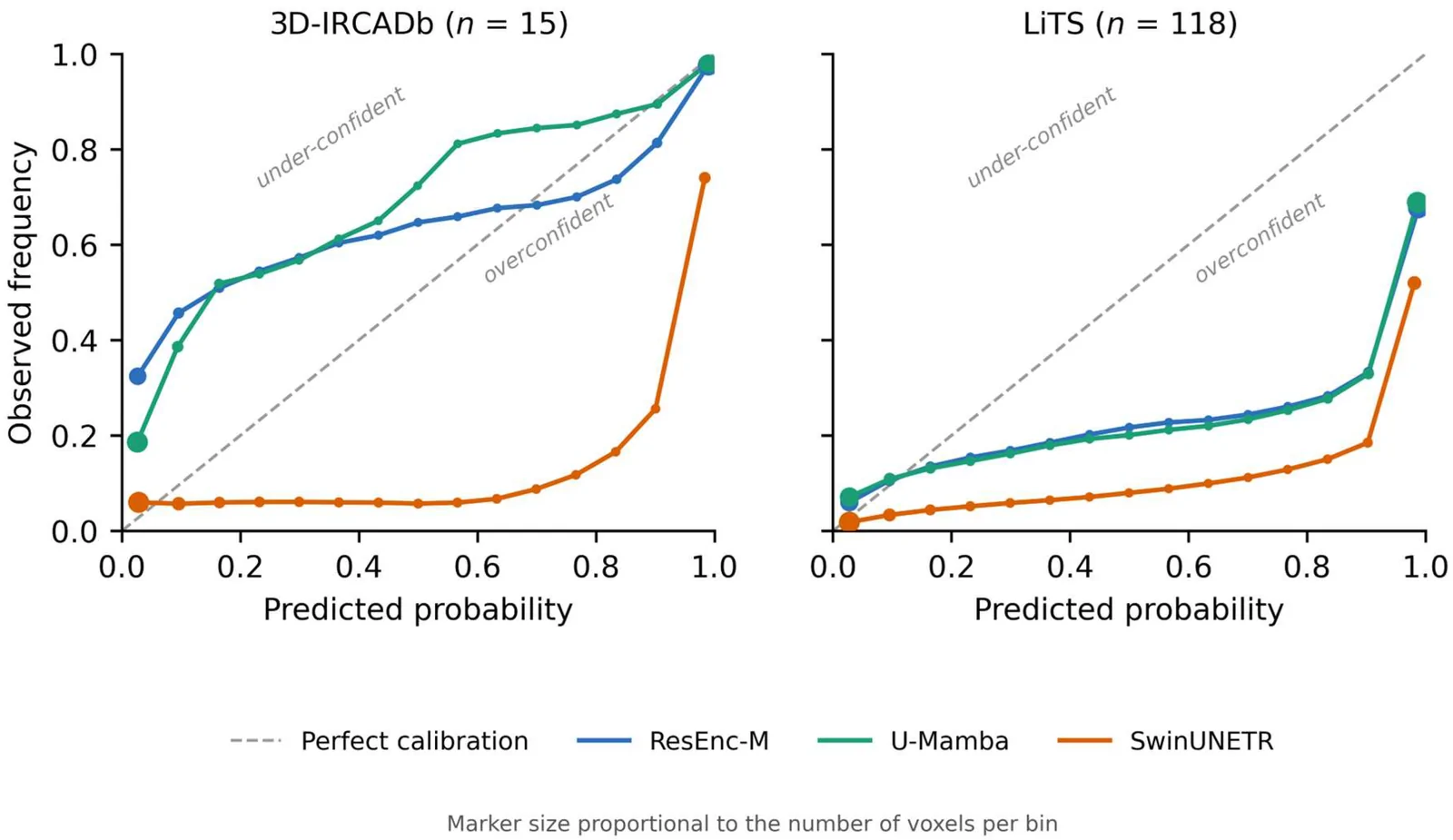
The reliability curves for both external cohorts, computed over voxels with predicted tumor probability >0.01. The marker area is proportional to the number of voxels in each bin. On LiTS all three models lie below the identity (overconfidence) and the convolutional and state-space curves are almost superimposed. On 3D-IRCADb those two models lie above the identity (underconfidence), showing that the direction of miscalibration depends on the target cohort. The transformer lies below the identity in both cohorts, and on 3D-IRCADb its observed tumor frequency remains flat across most of its output range.

Calibration error was substantial in both external cohorts, but the reliability curves show that its direction is not constant (Figure 4). On LiTS all three models lay below the diagonal, i.e., observed tumor frequency fell short of predicted probability across the entire range—classical overconfidence. On 3D-IRCADb, by contrast, the convolutional and state-space models lay above the diagonal over most of the range: they assigned low probabilities to voxels that in fact belonged to the tumor, i.e., they were underconfident. Because ECE is an unsigned quantity, the comparable IRCADb values (0.135–0.152) conceal this reversal; miscalibration direction is therefore a property of the target cohort rather than of the architecture, and ECE alone is insufficient to characterize it.

Two structural features of the reliability curves are worth noting. Voxel mass was concentrated almost entirely in the extreme bins (marker size in Figure 4), so intermediate bins are supported by comparatively few voxels and the mid-range behavior should be read with that in mind. On LiTS, the ResEnc-M and U-Mamba curves are nearly superimposed—the two architectures learned not merely similar case-level performance but near-identical voxel-level probability behavior.

The behavior of confidence during failure differed sharply between families (Table 10).

In cases where the convolutional and state-space models failed completely, their maximum predicted probability averaged 0.41–0.46—the models were, in effect, hesitant while wrong. SwinUNETR, by contrast, reached an average maximum probability of 0.977–0.981 in the cases it failed completely: it was maximally confident precisely where it was entirely wrong. This is consistent with its distance metrics (LiTS HD95 126 mm, roughly 30% higher than the other two) and with its failure profile: SwinUNETR produced no empty predictions at all on either cohort, yet produced zero-overlap predictions in 26 of 118 LiTS cases—it segmented confidently in the wrong location rather than abstaining. Case-level confidence summaries for all external cases are given in Table S5, and the bin-level reliability statistics underlying Figure 4 and the calibration errors in Table 10 are given in Table S6.

**Figure 5:**
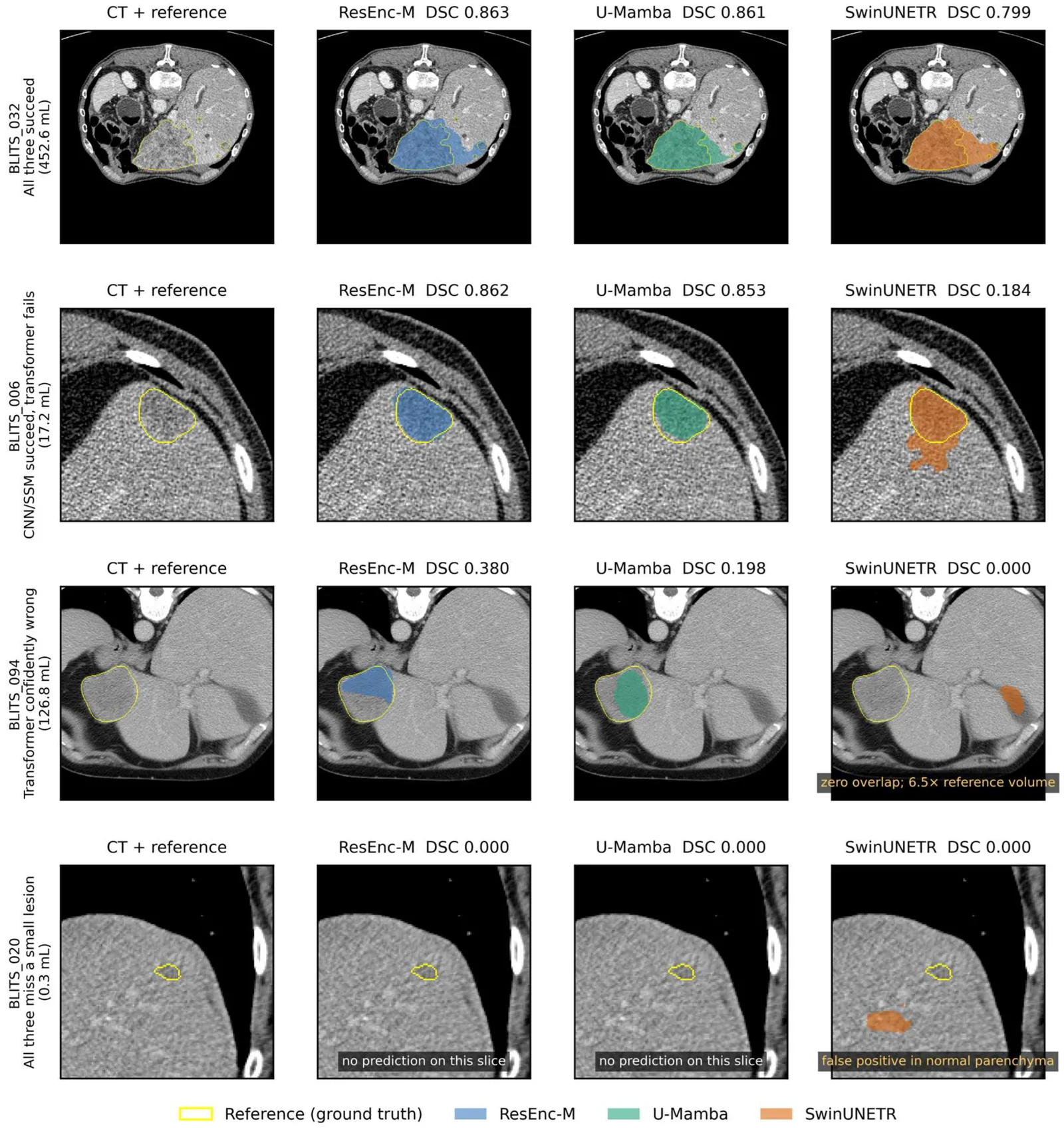
Representative LiTS cases illustrating four characteristic outcomes. Reference contours in yellow; model predictions filled. Row 1 (452.6 mL): all three architectures segment a large lesion accurately. Row 2 (17.2 mL): the convolutional and state-space models delineate the lesion closely, whereas the transformer covers the lesion but leaks well beyond the reference boundary into the adjacent parenchyma. Row 3 (126.8 mL): the transformer segmented 6.5 times the reference volume with zero overlap, placing its prediction over the spleen—202 pixels from the reference centroid on the same slice—at a maximum probability of 0.999. Row 4 (0.3 mL): all three miss a sub-centimeter lesion, but the failure modes differ—the convolutional and state-space models abstain, while the transformer places a confident false positive in normal parenchyma. Row 4 illustrates why complete failure must be defined both as empty prediction and as zero overlap.

Figure 5 shows this behavior directly. In a 126.8 mL lesion (Figure 5, row 3) the transformer produced no output at the tumor while reaching a maximum probability of 0.999 elsewhere; in a 0.3 mL lesion missed by all three models (row 4), the convolutional and state-space models abstained entirely whereas the transformer placed a confident false positive in normal parenchyma. The mechanism behind this behavior is visible in the IRCADb reliability curve (Figure 4, left): for SwinUNETR the observed tumor frequency remained flat at approximately 0.06 across predicted probabilities from 0.05 to 0.90, meaning that over most of its output range the predicted probability carried essentially no information about whether a voxel belonged to the tumor. The curve rises only in the topmost bin. SwinUNETR was also the only model whose curve lay below the diagonal in both cohorts, i.e., consistently overconfident irrespective of target cohort.

Across all architectures, case-level confidence nonetheless tracked segmentation quality (ρ = 0.726–0.857), which motivates the selective-prediction analysis below.

### 3.6. Confidence-Based Selective Prediction Substantially Improves Reliability

**Figure 6:**
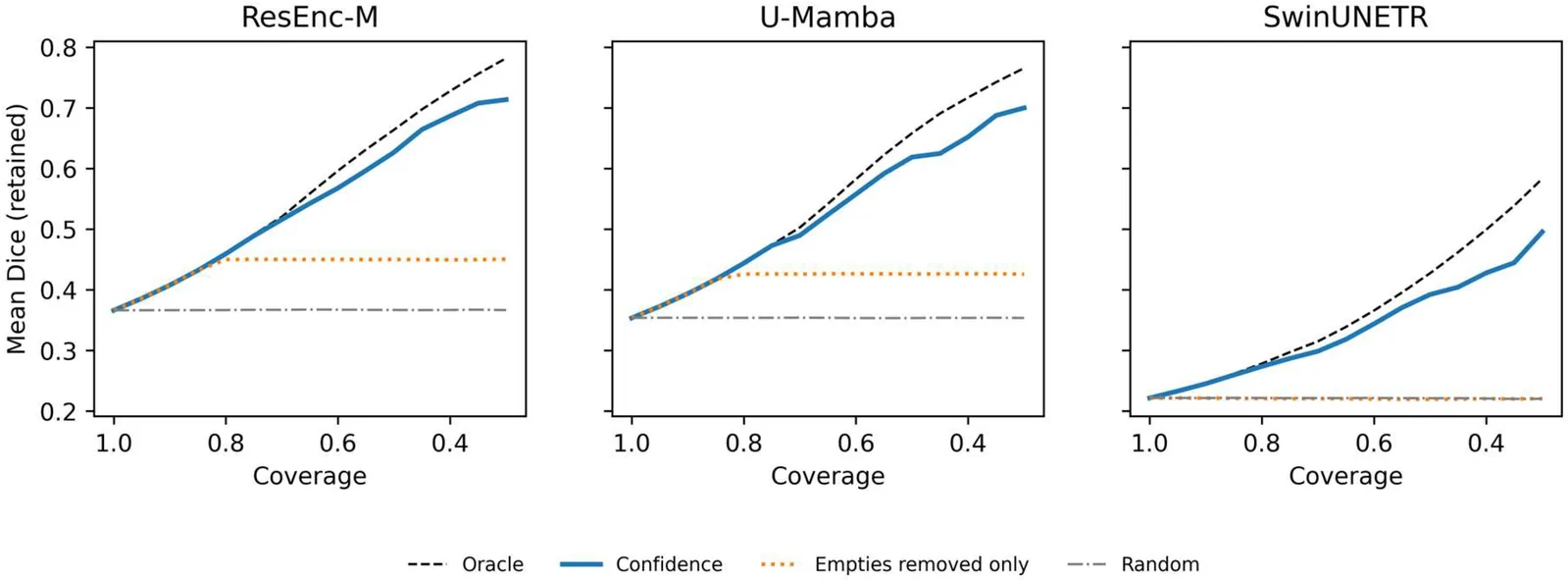
Risk–coverage behavior on LiTS (n = 118) for the three architectures. The cases are ranked by a selection score and the lowest-ranked are progressively deferred; the x-axis runs from full coverage on the left to 30% coverage on the right, and the y-axis is mean Dice on the retained cases. Solid: ranking by mean predicted confidence (empty predictions receive confidence zero and are deferred first). Dotted: empty predictions removed only, remaining cases retained in random order (mean of 1000 permutations). Dashed: oracle, cases deferred in ascending order of true Dice. Dash-dotted gray: random deferral (mean of 1000 permutations). Down to about 80% coverage the confidence curve coincides with the empties-only baseline; below that, confidence ranking continues to improve the retained subset and tracks the oracle closely for the convolutional and state-space models, whereas the transformer starts from a far lower level and gains least.

Deferring low-confidence cases improved performance on the retained subset monotonically (Table 11, Figure 6). Two components of this gain must be separated. Down to approximately 80% coverage, the confidence curve coincides with the baseline that simply removes the 22 empty predictions: excluding empty outputs alone raises mean Dice from 0.366 to 0.450, and confidence ranking adds nothing over this range. Below 80% coverage, however, confidence ranking continues to improve the retained subset while the empties-only baseline stays flat at 0.450: at 70% coverage Dice reached 0.516 (complete failures 28.8% → 7.2%); at 50% 0.627 with no complete failures; and at 40% 0.686, the internal cross-validation level. Over the whole curve, confidence ranking (AURC 0.456) approached the label-dependent oracle (0.436; excess AURC 0.020) and was clearly superior to random deferral (0.633) and to the empties-only baseline (0.562). The same pattern held for U-Mamba (AURC 0.473 vs. oracle 0.449). For SwinUNETR, which produced no empty predictions, confidence ranking was likewise better than random (0.667 vs. 0.779) but started from a far lower level, reaching only 0.392 at 50% coverage.

Deferral operates largely by removing small lesions. At 70% coverage the 35 deferred ResEnc-M cases had a median reference volume of 1.9 mL, 66% were below 5 mL, 22 were empty predictions, and their mean Dice was 0.01; the retained cases had a median volume of 27 mL, of which 17% were below 5 mL. Selective prediction therefore does not solve the small-lesion problem—it identifies the cases in which the model has failed and routes them, with the correct priority, back to the radiologist. Its value lies in making this failure visible rather than in recovering it. Two further observations follow. First, predicted confidence outperformed predicted lesion volume as a deferral criterion (LiTS selection-score ρ: 0.902 vs. 0.684 for ResEnc-M; 0.870 vs. 0.668 for U-Mamba), indicating that the model’s own uncertainty carries information beyond the simple heuristic ‘distrust small lesions’. Second, the mechanism was markedly less effective for SwinUNETR—at 50% coverage it reached only 0.392, against 0.62 for the other two—which follows directly from its confidence behavior during failure (Section 3.5). Calibration quality therefore determines not only how trustworthy a model’s probabilities are, but how well any downstream confidence-based safety mechanism can function.

### 3.7. Deferral Thresholds Transfer Between Cohorts

Because the coverage levels in Table 11 were evaluated on the same data used for reporting, we additionally tested whether an operating point selected on one external cohort transfers to the other. Confidence thresholds corresponding to 70% and 50% coverage were derived on 3D-IRCADb and applied unchanged to LiTS (Table 12).

For the convolutional and state-space models the transfer was well behaved: realized coverage on LiTS exceeded the target by 8–13 percentage points, and retained-subset performance improved substantially over the full cohort without any label-dependent tuning. For SwinUNETR the transfer failed in the operational sense—a threshold intended to retain 70% of cases retained only 40.7%—because its confidence distribution shifts markedly between cohorts. A deferral policy calibrated at one site would therefore reject far more studies than intended at another, at considerable cost in throughput.

### 3.8. The Cohorts Differ Most in the Property That Governs Performance

To characterize the distribution shift underlying these results, we compared acquisition and lesion characteristics across the three cohorts (Table 13).

The cohorts differ along several axes, but the largest discrepancy is precisely in the property identified in Section 3.4 as governing performance. Median lesion volume in the development cohort was 108.5 mL—roughly seven times that of either external cohort—and cases with total lesion volume below 5 mL, where all three models failed in 61–76% of instances, constituted 1.9% of the development cohort against 31.4% of LiTS. The models were therefore trained almost exclusively on the size regime in which they succeed and evaluated on cohorts containing a substantial fraction of the regime in which they fail.

Two further differences are consistent with the treatment-status mismatch: development tumors were denser (median 97.3 HU vs. 65.9 HU in LiTS), consistent with post-TACE lipiodol retention, and were typically solitary (median one lesion per case) whereas LiTS cases carried a median of five. Slice thickness also differed by a factor of 2.5, though all data were resampled to a common spacing before inference.

### 3.9. Anatomical Constraint Recovers Little, but Exposes an Arm-Specific Failure Pattern

Restricting predictions to the reference liver mask produced only modest gains (Table 14). On LiTS, Dice rose by 0.039–0.048 across the three architectures; on 3D-IRCADb, by 0.001–0.014. Complete-failure counts were unchanged in every architecture and cohort, confirming that the cases that failed entirely did so not because of extra-hepatic noise but because the lesion itself was not recognized. Since the liver mask was taken from the reference annotation, these values represent the benefit obtainable from a perfect liver segmentation; a realistic automated liver mask would yield less.

The final column reveals a difference the Dice values obscure. On LiTS, the convolutional and state-space models placed a median of 0.3% of their predicted tumor volume outside the liver, against 24.5% for the transformer—a difference of roughly two orders of magnitude in the typical case. For the former two the mean substantially exceeds the median (17.9% and 19.3% versus 0.3%), indicating anatomically clean predictions in most cases with occasional gross failures; for the transformer the median is itself high, indicating systematic extra-hepatic leakage rather than sporadic error. The case shown in Figure 5, row 3, where the transformer segmented the spleen, is therefore representative of its behavior rather than exceptional. At lesion level this manifests as 37 spurious components per case against fewer than one for the other two models (Table 9).

### 3.10. Combining Architectures Does Not Recover Performance

If the three families made independent errors, averaging their probability maps should improve on any single model. It did not: every pairwise and three-way combination performed below the best single model in both cohorts (Table 15).

The best combination fell 0.003 short of the best single model on 3D-IRCADb and 0.002 short on LiTS; the worst fell 0.023 short. Combining the two architectures that did not differ internally (ResEnc-M and U-Mamba) changed LiTS Dice from 0.366 to 0.364, i.e., contributed nothing, which is what the case-level concordance of ρ = 0.897 between them predicts. Adding the transformer degraded every combination it entered.

Ensembling averages away errors only when those errors are independent. Here they are not: the architectures fail on the same cases, so there is no complementary signal to exploit. This result is a direct practical consequence of the concordance reported in Section 3.3, and it parallels the anatomical analysis above: neither post-processing nor combination compensates for a shared failure mode.

### 3.11. Sensitivity Analysis: Pretrained Transformer Encoder

To test whether the transformer’s behavior reflects its initialization rather than its architecture, fold 0 was retrained with the Swin encoder initialized from self-supervised weights pretrained on 5050 CT volumes [32], with all other settings unchanged, and compared with the from-scratch fold-0 checkpoint under identical single-fold inference (Table 16, Figure S3). Pretraining did not improve the arm: internal validation Dice was 0.495 versus 0.511, LiTS Dice 0.181 (95% CI 0.142–0.224) versus 0.205 (0.158–0.249; paired difference −0.025, *p* < 0.0001), and 3D-IRCADb Dice 0.224 versus 0.214 (*p* = 0.75), with the same deficit relative to the convolutional and state-space fold-0 checkpoints (LiTS 0.314 and 0.308) in every volume stratum. The fragmented prediction pattern persisted and worsened (79 versus 56 false-positive components per LiTS case). The two transformer runs failed on the same cases (ρ = 0.94 on LiTS, 0.98 on 3D-IRCADb), and concordance with the convolutional model was unchanged (0.84 versus 0.84). Failure-time overconfidence was likewise unchanged: the pretrained arm assigned a mean confidence of 0.82 to its 21 complete failures versus 0.84 to its successes (from scratch: 0.82 versus 0.85), with maximum probabilities of 0.998 in both, and the confidence–Dice correlation that selective prediction relies on was weaker rather than stronger (ρ = 0.46 versus 0.60). The transformer arm’s external behavior is therefore a property of the architecture–regime combination rather than of its initialization; whether a substantially longer schedule would change it remains untested.

## 4. Discussion

### 4.1. Principal Findings

Three models spanning three architectural families, trained on identical data with identical splits, differed substantially on internal cross-validation but converged under cross-dataset transfer. On 3D-IRCADb the three did not differ significantly despite an internal spread of 0.21 Dice; on LiTS the convolutional and state-space models were formally equivalent while the transformer was significantly worse. In no comparison did the architecture with the strongest internal performance obtain an external advantage. Because optimizer, learning-rate schedule, mixed precision and deep supervision necessarily differed between arms (Table 1), each arm should be read as an architecture-plus-training-regime combination; the internal ranking does not isolate architecture. The external findings—concordant failure on the same cases—are unaffected by this, since concordance holds regardless of regime.

The concordance analysis explains why. Case-level Dice correlated at ρ = 0.836–0.964 across every architectural pairing, and largely the same cases failed across models. Two networks sharing no inductive bias beyond an encoder–decoder skeleton, trained with different optimizers, produced performance profiles that are near-interchangeable at the level of the individual patient—and, on LiTS, near-identical at the level of voxel-wise probability. Failure in this setting was largely a property of the case; residual architectural differences were confined to the transformer and were most visible on large lesions.

The property that governs it is lesion size. Volume predicted Dice in all six architecture × cohort combinations, with a monotone gradient from 0.03–0.10 Dice and 61–76% complete failure below 5 mL to 0.49–0.59 Dice and 2–7% failure above 50 mL. After adjustment for attenuation, lesion count, slice thickness and cohort, volume remained the only significant predictor, and its effect did not differ between architectures (Table 8); the same gradient was present at the level of individual lesions (Table 9).

### 4.2. Implications for How the Field Allocates Effort

If architectural substitution neither improves cross-dataset performance nor changes which cases fail, then the marginal return on architectural search—at least for this task, at this scale of training data—is low. Our results suggest that effort is better directed at the factors we found to be determinative: the diversity and size of the training distribution, and sensitivity to small lesions.

The development cohort is instructive in this regard. It comprises exclusively TACE-treated tumors, which exhibit lipiodol retention and post-treatment necrosis, whereas both external cohorts consist predominantly of untreated lesions. A model trained on this distribution learns appearance cues that are partly treatment-specific and partly institution-specific, and no architectural change can recover cues that were never represented in training. This is a data problem with a data solution: multi-institutional and multi-treatment-state training corpora, rather than deeper or more expressive networks.

The cohort comparison in Section 3.8 makes the mechanism concrete. The development cohort’s median lesion volume was approximately seven times that of either external cohort, and lesions below 5 mL—the regime in which every model failed most of the time—accounted for 1.9% of the development cases but 31.4% of the LiTS cases. The models were trained almost entirely within the size regime in which they succeed. Denser tumors (97.3 vs. 65.9 median HU, consistent with lipiodol retention) and predominantly solitary disease (one vs. five lesions per case) compound the mismatch. No architectural modification can compensate for a size regime that is essentially absent from training.

Nor can anatomical post-processing. Constraining predictions to a perfect liver mask—an upper bound unavailable in deployment—raised Dice by at most 0.048 and left the number of completely failed cases unchanged (Section 3.9). The deficit is not extra-hepatic noise that a region-of-interest step could remove; it is failure to recognize the lesion.

Nor does combining the families help. Every pairwise and three-way probability ensemble underperformed the best single model (Section 3.10). This follows directly from the concordance result: ensembling exploits error independence, and here the architectures err on the same cases. Architectural diversity, in this setting, is not diversity in any useful sense.

We emphasize that our finding is scoped. It does not imply that architecture is irrelevant in general—internal performance clearly differed, and with substantially larger and more diverse training corpora the ranking may well change. It implies that architecture did not determine generalization in this regime, and that internal benchmarking cannot be used to predict external behavior.

### 4.3. Calibration Determines Whether Safety Mechanisms Can Work

Miscalibration was substantial in both cohorts, but its direction was cohort-dependent: the convolutional and state-space models were overconfident on LiTS and underconfident on 3D-IRCADb. Since ECE is unsigned, it obscures this reversal, and we would recommend that studies reporting calibration under distribution shift present reliability curves rather than a single scalar.

The clinically consequential difference between architectures lay not in the magnitude of miscalibration but in how confidence behaved during failure. In completely failed cases, the convolutional and state-space models reached mean maximum probabilities of 0.41–0.46—they hedged while wrong. SwinUNETR reached 0.977–0.981: it was maximally confident precisely where it was entirely wrong, segmenting the wrong structure rather than abstaining, as reflected in its distance metrics and in its complete absence of empty predictions. Silent, confident error is the failure mode least likely to be caught in clinical use and should be weighted accordingly when models are selected for deployment; Figure 5 shows what this looks like at the level of an individual scan.

The anatomical analysis quantifies how systematic this is. On LiTS the transformer placed a median of 24.5% of its predicted tumor volume outside the liver, against 0.3% for the other two architectures. For the convolutional and state-space models the mean far exceeds the median, the signature of occasional gross errors against a background of anatomically plausible predictions; for the transformer the median is itself high, indicating that extra-hepatic leakage is its normal mode of operation rather than an outlier. A model that routinely assigns tumor labels outside the target organ, with high confidence, is poorly suited to unsupervised deployment irrespective of its mean Dice.

This distinction has a direct operational consequence. Automated quality control and staged clinical validation are established components of safe deployment [36,37]. Because confidence tracked segmentation quality (ρ = 0.726–0.857), deferring low-confidence studies to expert review improved the retained subset monotonically: for the best model on LiTS, deferring 30% of cases raised Dice from 0.366 to 0.516 and reduced complete failures from 28.8% to 7.2%, and deferring 60% restored performance to the internal cross-validation level—of which the first 0.08 is obtained merely by discarding empty outputs, and the remainder by the ranking itself. The deferred cases are predominantly sub-5 mL lesions, so the mechanism triages rather than resolves the hardest cases. But the mechanism worked far less well for the model with the worst failure-time calibration, which reached only 0.392 even at 50% coverage. Calibration quality thus governs not only the interpretability of a model’s probabilities but the achievable performance of any confidence-based safety layer built on top of it.

Predicted confidence also outperformed predicted lesion volume as a deferral criterion, indicating that the model’s own uncertainty encodes more than the heuristic ‘distrust small lesions’. Both criteria are computable without reference labels and are therefore deployable.

Threshold transfer results support the practical feasibility of this approach but also delimit it. For the convolutional and state-space models, a threshold derived on 3D-IRCADb and applied unchanged to LiTS retained 78% and 83% of cases respectively and raised Dice from 0.366 to 0.469 and from 0.354 to 0.426—an improvement obtained without access to external labels. For SwinUNETR the same procedure retained only 40.7% of cases against a 70% target, since its confidence distribution shifts substantially between cohorts. A confidence-based safety layer is therefore transferable in principle, but the transferability of its operating point is itself model-dependent and must be verified rather than assumed.

### 4.4. Relation to Existing Work

Our internal ranking is consistent with the broader literature. Convolutional baselines remain highly competitive with transformer alternatives on medical segmentation datasets of moderate size, and transformer models are comparatively data-hungry: Swin UNETR required self-supervised pretraining on 652 unlabeled volumes before surpassing nnU-Net on lesion segmentation [8], and transformer segmentation networks are additionally demanding at inference [7]. With 103 training patients, the transformer deficit we observed is the expected regime rather than an anomaly. The contribution here is not the internal ranking but the demonstration that this ranking does not survive transfer, and that failure is concordant at the case level across families.

The magnitude of our generalization gap is in line with independent reports. In colorectal liver metastases, per-case Dice fell from 0.80 internally to 0.60 externally [16]; in HCC treated with TACE—the closest prior setting to ours—a multi-task model reported 0.736 Dice with an external test cohort [17]; and CT-based liver-lesion models are typically validated on external cohorts of only a few dozen patients [38], as is the case for our 3D-IRCADb cohort. Our drop is steeper, consistent with a development cohort restricted to a single treatment state.

Our central claim—that architecture does not determine transfer—has an independent parallel outside the liver. Comparing three architectures across unseen cardiac MRI domains, the simplest model proved the most generalizable [39], and in abdominal CT classification both convolutional and transformer models degraded by a similar margin externally [20]. The most instructive parallel is a liver cancer histopathology model whose internal AUC of 0.998 collapsed to 0.745 externally and recovered only once external data were added to training [19]: the remedy was data, not architecture. Our results extend this pattern to volumetric segmentation and, through case-level concordance, establish it at the level of individual patients rather than cohort averages.

Our findings should be read against the Medical Segmentation Decathlon, which concluded that state-of-the-art segmentation algorithms generalize well to unseen tasks [29]. That conclusion concerns generalization after retraining on the new task. The complementary case—transfer without any adaptation, which is what deployment at a new institution actually entails—is the one we tested, and there the conclusion does not hold. The two results are compatible and, taken together, delineate where the burden lies: on acquiring representative data rather than on the algorithm.

Finally, the size dependence we observed is consistent with the long-standing difficulty of small-lesion detection, and is not unique to the transfer setting. Even in-domain, the best LiTS submissions achieved lesion-wise recall of only 0.458–0.554 [28]. Our stratified estimates quantify the effect under genuine cross-dataset conditions and show it to be architecture-invariant.

### 4.5. How Much of the Gap Is Domain Shift, and How Much Is Task Difficulty?

An external Dice of 0.366 is only interpretable against the performance attainable on the same cohort by models that were trained on it. The LiTS benchmark provides that reference: across three challenge events, 75 submitted algorithms trained on LiTS data reached best tumor Dice values of 0.674, 0.702, and 0.739 on the LiTS test partition. In-domain liver-tumor segmentation on this cohort therefore plateaus at roughly 0.70, well below the near-0.95 values typical of organ segmentation, confirming that the task is intrinsically difficult. Our best cross-dataset result of 0.366 sits at approximately half that ceiling, which localizes the majority of the deficit to distribution shift rather than to task impossibility—and, correspondingly, indicates that a substantial portion is in principle recoverable through better-matched training data.

The same benchmark also corroborates our size finding from the opposite direction. Even for in-domain models, the best lesion-wise recall reported was 0.458 to 0.554, meaning that roughly half of all lesions were missed by the strongest algorithms evaluated on data drawn from their own training distribution. Small-lesion sensitivity is thus a limitation of the task itself, which cross-dataset transfer amplifies but does not create. This reinforces our conclusion that small-lesion detection warrants a dedicated research effort rather than being treated as a byproduct of general segmentation accuracy.

Notably, the selective-prediction results converge on the same ceiling from below: at 40% coverage our best model reached 0.686 on retained LiTS cases, essentially the in-domain plateau reported for the benchmark. Deferral does not exceed what the task allows; it recovers the achievable fraction of it.

### 4.6. Limitations

Several limitations qualify these findings.

First, the external task is not HCC segmentation in the strict sense: LiTS and 3D-IRCADb contain lesions of mixed and unlabeled etiology, so the external shift combines domain, disease-type and annotation-protocol differences, and an HCC-only subset could not be isolated. Our conclusions concern the transfer of a model trained on treated HCC to general liver-tumor segmentation.

The development cohort comprised 103 patients from a single collection with a homogeneous treatment state, which limits both absolute performance and the generality of the conclusion. Annotation cost is the usual reason such cohorts remain small [40]; a larger, multi-institutional development set might alter the architectural ranking.

Training regimes were not fully identical across arms. U-Mamba required mixed precision to be disabled with a reduced learning rate, and SwinUNETR was trained with AdamW and without deep supervision under its reference configuration. We considered these deviations preferable to imposing a single regime that would disadvantage particular architectures, but they mean that the internal differences reported here reflect an architecture-plus-regime combination rather than architecture alone. The central conclusion is robust to this caveat, since it concerns concordance of failure and the absence of external advantage rather than the precise internal ranking. The transformer arm was trained from scratch under a 250-epoch cosine schedule; validation pseudo-Dice had plateaued in folds 0, 2 and 3, was still rising slowly in fold 1, and remained low and unstable in fold 4 (Figure S2), so part of its internal deficit—and possibly its failure-time overconfidence and spurious components—may reflect undertraining rather than architecture. A one-fold sensitivity analysis with a self-supervised pretrained encoder did not change the transformer’s external performance, its fragmented prediction pattern or its failure-time overconfidence (Section 3.11); these findings are therefore unlikely to be artifacts of initialization, although a longer training schedule was not tested. Transformer-specific findings should nevertheless be read as arm-specific.

3D-IRCADb contains only 15 tumor-bearing cases, so all estimates from that cohort carry wide confidence intervals; the primary statistical weight rests on LiTS. In particular, the 3D-IRCADb cohort permits neither equivalence nor a difference to be established for the transformer (Table 4); only the LiTS results are conclusive for that arm. Cohort sizes were fixed by data availability rather than chosen by power calculation. At fold level the design is particularly constrained: with five folds, 0.063 is the smallest *p*-value the Wilcoxon signed-rank test can return, so the internal post hoc comparisons cannot reach a Bonferroni-corrected threshold regardless of effect size, and we therefore interpret them through effect sizes and the consistency of ranking across folds rather than through significance alone.

The selective-prediction analysis reports risk–coverage behavior rather than a prospectively validated operating threshold. Coverage levels in Table 11 were evaluated on the same external data used for reporting and should be regarded as an upper bound; the threshold transfer experiment (Table 12) mitigates but does not eliminate this concern, since it uses only two cohorts and cannot establish how thresholds behave across a wider range of sites. Moreover, deferral removes predominantly small lesions (Section 3.6), so it triages rather than resolves the hardest cases. Reverse-direction training (LiTS → HCC-TACE-Seg) and small-lesion oversampling, which would test the volume effect under a different training distribution, were beyond the scope of this study and are noted as future work.

Finally, we did not train an upper-bound reference model on the external cohorts themselves. We instead used published in-domain benchmark performance as the ceiling (Section 4.5), which is adequate for the comparison drawn here but is not matched to our preprocessing, evaluation code, or case subset; a locally trained oracle would give a tighter bound.

## 5. Conclusions

For liver-tumor segmentation models trained on a single, homogeneous, TACE-treated HCC cohort (n = 103) and transferred without adaptation to mixed-etiology external cohorts, cross-dataset failure was determined primarily by lesion size, not by architectural family: the convolutional and state-space models were formally equivalent and failed on the same cases, while the transformer trailed both on LiTS. All three degraded severely on two independent external cohorts, and the architecture with the best internal performance conferred no external advantage.

Two practical implications follow. First, internal cross-validation is not a valid basis for predicting external behavior, and architectural benchmarking on a single cohort should not be treated as evidence of transferability. Second, because confidence tracked segmentation quality, confidence-based selective prediction offers a deployable mitigation that improves reliability on retained cases without any retraining—but its effectiveness depends on how a model behaves when it fails, which did differ between arms. Improving cross-institutional reliability in this task is likely to require broader training distributions, explicit attention to small lesions, and calibration-aware safety mechanisms rather than further architectural substitution.

## Figures and Tables

**Table 1:** The architectural families and training configurations of the three compared models. All arms were trained on the same development cohort (n = 103) with identical patient-disjoint five-fold splits, an identical 250-epoch budget, and identical patch size, loss (compound Dice + cross-entropy), sampling, and augmentation. Deviations were limited to mixed precision, optimizer, learning-rate schedule and deep supervision as shown. LR, learning rate; SGD, stochastic gradient descent; SSM, state-space model.

Model	Family	Plans	Optimizer	LR Schedule	Mixed Precision	Deep Supervision
nnU-Net ResEnc-M	Convolutional (residual encoder)	‘nnUNetResEncUNetMPlans’	SGD (momentum 0.99, Nesterov)	poly, lr_0_ = 0.01	enabled	yes (5 scales)
U-Mamba Bot	State-space (SSM)	‘nnUNetPlans’	SGD (momentum 0.99, Nesterov)	poly, lr_0_ = 1 × 10^−3^	disabled	yes (5 scales)
SwinUNETR (feature size 48)	Transformer	‘nnUNetPlans’	AdamW (weight decay 0.01)	cosine, lr_0_ = 8 × 10^−4^, η_min = 1 × 10^−6^	disabled	no

**Table 2:** Internal five-fold cross-validation Dice per architecture.

Fold	ResEnc-M (CNN)	U-Mamba Bot (SSM)	SwinUNETR (Transformer)
0	0.696	0.638	0.511
1	0.714	0.667	0.519
2	0.570	0.599	0.435
3	0.723	0.725	0.584
4	0.724	0.682	0.344
Mean ± SD	0.685 ± 0.065	0.662 ± 0.044	0.479 ± 0.093

**Table 3:** External performance and generalization gap.

		ResEnc-M	U-Mamba	SwinUNETR
Internal	DSC	0.685	0.662	0.479
3D-IRCADb (n = 15)	DSC [95% CI]	0.260 [0.116–0.431]	0.272 [0.125–0.441]	0.255 [0.113–0.417]
	IoU	0.194 [0.080–0.333]	0.202 [0.086–0.336]	0.185 [0.078–0.311]
	HD95 (mm)	62.7 [46.7–76.8]	49.8 [31.8–68.4]	91.2 [70.5–112.3]
	ASSD (mm)	28.5 [18.7–38.4]	21.0 [12.4–29.8]	42.2 [26.2–60.2]
	Empty predictions	4/15	5/15	0/15
	Δ	−0.425	−0.390	−0.224
LiTS (n = 118)	DSC [95% CI]	0.366 [0.305–0.424]	0.354 [0.293–0.412]	0.222 [0.175–0.270]
	IoU	0.279 [0.228–0.329]	0.268 [0.218–0.316]	0.154 [0.118–0.190]
	HD95 (mm)	104.8 [85.0–125.4]	99.1 [80.1–117.6]	126.3 [111.7–142.0]
	ASSD (mm)	48.1 [33.8–63.6]	52.9 [37.6–69.1]	57.3 [49.2–66.1]
	Empty predictions	22/118	20/118	0/118
	Δ	−0.319	−0.309	−0.257

**Table 4:** Equivalence testing of paired case-level Dice differences (pre-specified margin ±0.05; paired bootstrap, 5000 resamples). Equivalence is declared when the 90% CI lies entirely within the margin. Wilcoxon *p*-values compared against the Bonferroni threshold α = 0.0167.

Cohort/Stratum	Pair	n	Mean Δ Dice [90% CI]	Verdict	Wilcoxon *p*
3D-IRCADb	CNN–SSM	15	−0.012 [−0.039, 0.017]	Equivalent	0.374
	CNN–Transformer	15	0.005 [−0.043, 0.058]	Inconclusive	0.938
	SSM–Transformer	15	0.017 [−0.012, 0.048]	Equivalent	0.695
LiTS	CNN–SSM	118	0.013 [−0.011, 0.035]	Equivalent	0.184
	CNN–Transformer	118	0.145 [0.112, 0.177]	Transformer inferior	<0.0001
	SSM–Transformer	118	0.132 [0.103, 0.163]	Transformer inferior	<0.0001
Pooled, <5 mL	CNN–SSM	38	0.009 [−0.040, 0.058]	Inconclusive	0.594
	CNN–Transformer	38	0.076 [0.030, 0.127]	Transformer inferior	0.011
	SSM–Transformer	38	0.067 [0.027, 0.111]	Transformer inferior *	0.027
Pooled, 5–20 mL	CNN–SSM	36	0.019 [−0.023, 0.056]	Inconclusive	0.137
	CNN–Transformer	36	0.198 [0.133, 0.265]	Transformer inferior	0.0001
	SSM–Transformer	36	0.179 [0.112, 0.250]	Transformer inferior	0.0009
Pooled, 20–50 mL	CNN–SSM	15	0.006 [−0.057, 0.078]	Inconclusive	0.551
	CNN–Transformer	15	0.190 [0.112, 0.272]	Transformer inferior	0.0026
	SSM–Transformer	15	0.184 [0.108, 0.261]	Transformer inferior	0.0020
Pooled, >50 mL	CNN–SSM	44	0.004 [−0.017, 0.025]	Equivalent	0.750
	CNN–Transformer	44	0.097 [0.052, 0.140]	Transformer inferior	0.0008
	SSM–Transformer	44	0.093 [0.058, 0.130]	Transformer inferior	0.0001

* *p* above the Bonferroni threshold but CI excludes zero.

**Table 5:** Case-level Spearman correlation of Dice between architectures.

Cohort	Pair	ρ Raw (Ensembles)	ρ Partial|Log Volume [95% CI]	ρ Same-Fold Cross-Arch (Mean, Range)	ρ Within-Arch Reference (Mean, Range)
3D-IRCADb	CNN–SSM	0.964	0.960 [0.800–1.000]	0.867 (0.78–0.98)	CNN 0.890 (0.84–0.94)
	CNN–Transformer	0.858	0.788 [0.433–0.941]	0.782 (0.67–0.90)	SSM 0.849 (0.76–0.92)
	SSM–Transformer	0.924	0.840 [0.482–0.960]	0.860 (0.76–0.91)	Transformer 0.940 (0.90–0.97)
LiTS	CNN–SSM	0.897	0.807 [0.691–0.887]	0.863 (0.79–0.90)	CNN 0.900 (0.86–0.93)
	CNN–Transformer	0.836	0.672 [0.532–0.786]	0.822 (0.79–0.84)	SSM 0.877 (0.82–0.91)
	SSM–Transformer	0.872	0.731 [0.622–0.818]	0.849 (0.80–0.89)	Transformer 0.930 (0.91–0.95)

Partial correlations: ranks residualized on log10 reference lesion volume. Within-architecture reference: mean pairwise ρ between the five single-fold checkpoints of the same architecture. Same-fold cross-architecture: mean over folds k = 0–4 of ρ between the fold-k checkpoints of two architectures. Ensemble values are between the five-fold ensembles used throughout the paper.

**Table 6:** Spearman correlation between reference lesion volume and Dice.

Cohort	ResEnc-M	U-Mamba	SwinUNETR
3D-IRCADb	0.587 (*p* = 0.021)	0.708 (*p* = 0.0031)	0.778 (*p* = 0.00064)
LiTS	0.673 (*p* = 7.3 × 10^−17^)	0.698 (*p* = 1.7 × 10^−18^)	0.788 (*p* = 3.7 × 10^−26^)

**Table 7:** Performance stratified by reference lesion volume (both external cohorts pooled, n = 133). Complete failure defined as zero overlap.

Volume	n	DSC ResEnc-M	DSC U-Mamba	DSC SwinUNETR	Fail ResEnc-M	Fail U-Mamba	Fail SwinUNETR
<5 mL	38	0.101	0.091	0.025	68.4%	76.3%	60.5%
5–20 mL	36	0.298	0.279	0.100	27.8%	36.1%	13.9%
20–50 mL	15	0.439	0.433	0.249	13.3%	6.7%	0.0%
>50 mL	44	0.590	0.587	0.494	6.8%	2.3%	2.3%

Lesions below 5 mL were completely missed in 61–76% of cases, versus 2–7% for lesions above 50 mL—a roughly tenfold difference in failure rate and a five- to twentyfold difference in mean Dice.

**Table 8:** Mixed-effects linear model of case-level Dice (399 observations = 133 cases × 3 architectures; random intercept per case; maximum likelihood). Continuous covariates standardized (coefficients = Dice change per SD). Reference levels: 3D-IRCADb, ResEnc-M.

Term	Coefficient [95% CI]	*p*
log10 lesion volume (per SD)	0.210 [0.168, 0.252]	<0.001
Mean tumor HU (per SD)	−0.014 [−0.051, 0.023]	0.46
Within-tumor HU SD (per SD)	−0.028 [−0.067, 0.012]	0.17
log10 lesion count (per SD)	−0.003 [−0.044, 0.038]	0.89
Slice thickness (per SD)	0.000 [−0.037, 0.038]	0.99
Cohort: LiTS vs. 3D-IRCADb	0.088 [−0.028, 0.203]	0.14
Architecture: U-Mamba vs. ResEnc-M	−0.010 [−0.041, 0.021]	0.54
Architecture: SwinUNETR vs. ResEnc-M	−0.129 [−0.160, −0.098]	<0.001
Case random-intercept variance/residual	0.037/0.017 (ICC 0.69)	
AIC: full/volume-only/no-volume	−205.5/−211.2/−135.5	
Volume × architecture interaction	U-Mamba 0.001 (*p* = 0.96); SwinUNETR −0.016 (*p* = 0.30)	

**Table 9:** Lesion-wise detection on the external cohorts, stratified by the volume of each individual reference lesion (detected: IoU ≥ 0.1 with the union of overlapping predicted components). False positives: predicted connected components with no overlap with any reference lesion.

Cohort	Stratum	n Lesions	ResEnc-M	U-Mamba	SwinUNETR	Any-Overlap (ResEnc/U-Mamba/Swin)
LiTS	<1 mL	565	0.032	0.028	0.021	0.21/0.16/0.30
	1–5 mL	160	0.269	0.244	0.175	0.47/0.43/0.53
	5–20 mL	110	0.591	0.555	0.400	0.76/0.72/0.83
	>20 mL	73	0.808	0.795	0.603	0.92/0.93/0.97
	all	908	0.204	0.192	0.141	0.38/0.34/0.46
	FP components/case, mean (median)		0.68 (0)	1.02 (0)	37.3 (16.5)	
3D-IRCADb	<1 mL	57	0.000	0.000	0.035	0.02/0.02/0.09
	1–5 mL	33	0.182	0.091	0.152	0.24/0.15/0.24
	5–20 mL	17	0.176	0.235	0.118	0.24/0.35/0.47
	>20 mL	10	0.600	0.600	0.700	0.60/0.80/1.00
	all	117	0.128	0.111	0.137	0.16/0.17/0.27
	FP components/case, mean (median)		0.40 (0)	0.40 (0)	60.3 (8)	

**Table 10:** Calibration and confidence behavior.

Cohort	Metric	ResEnc-M	U-Mamba	SwinUNETR
3D-IRCADb	ECE	0.152	0.135	0.202
	MCE	0.361	0.354	0.668
	Max probability in failed cases	0.459	0.280	0.981
	Confidence ↔ Dice (ρ)	0.844	0.782	0.857
LiTS	ECE	0.222	0.219	0.216
	MCE	0.571	0.574	0.718
	Mean confidence within prediction	0.842	0.842	0.793
	Max probability in failed cases	0.408	0.443	0.977
	Confidence ↔ Dice (ρ)	0.852	0.825	0.726

**Table 11:** Risk–coverage behavior on LiTS (n = 118); cases ranked by mean predicted confidence. The values are mean DSC on the retained subset (complete-failure rate, %). Two reference curves are shown for ResEnc-M: the removal of empty predictions only (remaining cases in random order, mean of 1000 permutations) and an oracle that defers cases in ascending order of true DSC. AURC, area under the risk–coverage curve over 30–100% coverage (risk = 1 − DSC; lower is better).

Coverage	n	DSC ResEnc-M (Fail %)	DSC U-Mamba (Fail %)	DSC SwinUNETR (Fail %)	ResEnc-M, Empties Removed Only	ResEnc-M, Oracle
100%	118	0.366 (28.8)	0.354 (32.2)	0.222 (22.0)	0.366	0.366
90%	106	0.408 (20.8)	0.394 (24.5)	0.245 (19.8)	0.408	0.408
80%	94	0.459 (11.7)	0.444 (16.0)	0.274 (17.0)	0.450	0.460
70%	83	0.516 (7.2)	0.490 (12.0)	0.299 (15.7)	0.450	0.521
60%	71	0.568 (2.8)	0.558 (7.0)	0.344 (11.3)	0.450	0.596
50%	59	0.627 (0.0)	0.619 (1.7)	0.392 (6.8)	0.450	0.664
40%	47	0.686 (0.0)	0.652 (2.1)	0.428 (4.3)	0.449	0.728
30%	35	0.713 (0.0)	0.700 (0.0)	0.495 (2.9)	0.451	0.782
AURC		0.456	0.473	0.667	0.562	0.436

Random deferral AURC: 0.633 (ResEnc-M), 0.646 (U-Mamba), 0.779 (SwinUNETR). Oracle AURC: 0.436, 0.449, 0.638. Full curves for all arms are given in Table S7.

**Table 12:** Threshold transfer: operating point selected on 3D-IRCADb, applied to LiTS.

Model	Target Coverage	Threshold	Realized Coverage on LiTS	DSC on Retained	Complete Failure
ResEnc-M	70%	0.548	78.0%	0.469 (vs. 0.366 overall)	10.9%
ResEnc-M	50%	0.736	66.1%	0.536	5.1%
U-Mamba	70%	0.104	83.1%	0.426 (vs. 0.354)	18.4%
U-Mamba	50%	0.701	69.5%	0.496	11.0%
SwinUNETR	70%	0.802	40.7%	0.426 (vs. 0.222)	4.2%
SwinUNETR	50%	0.822	32.2%	0.476	2.6%

**Table 13:** Cohort characteristics; median [IQR] unless stated.

Characteristic	HCC-TACE-Seg (Development, n = 103)	3D-IRCADb (n = 15)	LiTS (n = 118)
Slice thickness (mm)	2.5 [2.5–2.5]	1.6 [1.6–1.8]	1.0 [0.8–2.0]
In-plane spacing (mm)	0.781	0.736	0.758
Tumor HU (mean)	97.3 [82.5–111.3]	76.2 [66.2–82.5]	65.9 [51.4–79.1]
Within-tumor HU SD	31.3 [22.6–37.2]	29.5 [26.4–37.0]	27.5 [22.7–32.5]
Lesion volume (mL)	108.5 [37.5–378.6]	13.8 [7.2–99.8]	16.6 [3.5–108.3]
Lesions per case	1 (1,3)	2 (1–3,6–8,25,26)	5 (1–3,6–8,10–12,25,26)
Proportion of cases <5 mL	1.9%	6.7%	31.4%

**Table 14:** The effect of constraining predictions to the reference liver mask.

Cohort	Model	DSC Unconstrained	DSC Liver-Constrained	Δ	Complete Failures	Predicted Volume Outside Liver, Mean (Median)
3D-IRCADb	ResEnc-M	0.260	0.274	+0.014	7 → 7	12.9% (0.0%)
	U-Mamba	0.272	0.273	+0.001	6 → 6	0.4% (0.0%)
	SwinUNETR	0.255	0.261	+0.006	3 → 3	16.5% (4.7%)
LiTS	ResEnc-M	0.366	0.410	+0.044	34 → 34	17.9% (0.3%)
	U-Mamba	0.354	0.392	+0.039	37 → 37	19.3% (0.3%)
	SwinUNETR	0.222	0.270	+0.048	26 → 26	36.4% (24.5%)

**Table 15:** Probability-map ensembles versus single models (Dice; complete failures in parentheses).

Combination	3D-IRCADb	LiTS
ResEnc-M alone	0.260 (7)	0.366 (34)
U-Mamba alone	0.272 (6)	0.354 (37)
SwinUNETR alone	0.255 (3)	0.222 (26)
ResEnc-M + U-Mamba	0.269 (6)	0.364 (36)
ResEnc-M + SwinUNETR	0.264 (6)	0.353 (34)
U-Mamba + SwinUNETR	0.266 (5)	0.343 (39)
All three	0.264 (6)	0.358 (35)

**Table 16:** Sensitivity analysis: fold-0 SwinUNETR trained from scratch versus with a self-supervised pretrained encoder, compared with the fold-0 convolutional and state-space checkpoints. All values are single-fold inference without test-time augmentation and are therefore lower than the five-fold-ensemble results in Table 3; they are comparable only within this table. Confidence metrics on LiTS.

	Swin, from Scratch	Swin, Pretrained Encoder	ResEnc-M	U-Mamba
Internal fold-0 validation Dice	0.511	0.495	—	—
LiTS Dice, mean [95% CI]	0.205 [0.158–0.249]	0.181 [0.142–0.224]	0.314 [0.262–0.369]	0.308 [0.254–0.368]
LiTS Dice by stratum: <5/5–20/20–50/>50 mL	0.011/0.076/0.258/0.480	0.009/0.061/0.200/0.439	0.060/0.264/0.362/0.589	0.052/0.250/0.356/0.589
LiTS zero-overlap cases, %	22.0	17.8	24.6	30.5
LiTS empty predictions, %	0.0	0.0	1.7	5.1
LiTS false-positive components/case	55.5	79.2	3.4	5.1
3D-IRCADb Dice, mean [95% CI]	0.214 [0.088–0.358]	0.224 [0.099–0.358]	0.248 [0.097–0.400]	0.276 [0.121–0.440]
3D-IRCADb false-positive components/case	26.6	52.1	1.3	3.7
ρ with ResEnc-M fold 0 (LiTS/3D-IRCADb)	0.844/0.903	0.838/0.870	—	0.899/0.979
ρ with Swin from scratch (LiTS/3D-IRCADb)	—	0.935/0.979	0.844/0.903	0.886/0.914
Mean confidence, complete failures/successes	0.820/0.847	0.819/0.839		
Mean max probability on complete failures	0.998	0.998		
Spearman ρ (confidence, Dice)	0.595	0.462		
Dice at 70%/50% coverage	0.270/0.343	0.232/0.286		
Paired Swin-pt − Swin-scratch	LiTS −0.025 (*p* < 0.0001); 3D-IRCADb +0.010 (*p* = 0.75)			

## Data Availability

This study analyzed exclusively publicly available data; no new data were created. The development cohort (HCC-TACE-Seg) is available from The Cancer Imaging Archive at https://doi.org/10.7937/TCIA.5FNA-0924 (accessed on 14 September 2026) under a Creative Commons Attribution 4.0 International license [23,24]. The 3D-IRCADb-01 database is available from IRCAD at https://www.ircad.fr/research/data-sets/liver-segmentation-3d-ircadb-01/ (accessed on 14 September 2026) under a Creative Commons Attribution–NonCommercial–NoDerivatives 4.0 International license [27]. The Medical Segmentation Decathlon Task03 (Liver) dataset, which derives from the Liver-Tumor Segmentation Benchmark, is available at http://medicaldecathlon.com/ (accessed on 14 September 2026) [28,29]. The analysis code, the exact cross-validation fold assignment (splits_final.json), the per-case results underlying all tables and figures, and the scripts used to generate them, including all sensitivity analyses, are available at https://github.com/huseyinKutlu/hcc-crossdataset-segmentation (accessed on 1 October 2026) and archived at Zenodo (version DOI 10.5281/zenodo.23090049; concept DOI 10.5281/zenodo.23090048, accessed on 1 October 2026). Trained model checkpoints are available from the corresponding author upon reasonable request.

## Supplementary Materials

The following supporting information can be downloaded at https://www.mdpi.com/article/10.3390/cancers18203362/s1, Table S1: Completed CLAIM 2024 checklist; Table S2: Completed TRIPOD+AI checklist; Table S3: Training configuration and computational environment; Table S4: Case-level performance for the three architectures; Table S5: Case-level confidence behavior; Table S6: Reliability-curve bin statistics; Table S7: Risk–coverage curves for all architectures, with reference curves; Table S8: Lesion-wise detection per cohort, architecture and lesion-size stratum; Table S9: Within-architecture reference and same-fold cross-architecture concordance; Figure S1: Flow of cases through the three cohorts; Figure S2: Training dynamics of the transformer arm; Figure S3: Fold-0 training dynamics, from-scratch versus pretrained encoder. References [32,33,34,35] are cited in the Supplementary Materials.

## Abbreviations

The following abbreviations are used in this manuscript:

AI	artificial intelligence
ASSD	average symmetric surface distance
AUC	area under the receiver operating characteristic curve
CLAIM	Checklist for Artificial Intelligence in Medical Imaging
CNN	convolutional neural network
CT	computed tomography
DSC	Dice similarity coefficient
ECE	expected calibration error
HCC	hepatocellular carcinoma
HD95	95th-percentile Hausdorff distance
IoU	intersection over union
IQR	interquartile range
LiTS	Liver-Tumor Segmentation Benchmark
MCE	maximum calibration error
MSD	Medical Segmentation Decathlon
SGD	stochastic gradient descent
SSM	state-space model
TACE	transarterial chemoembolization
TCIA	The Cancer Imaging Archive
TRIPOD+AI	Transparent Reporting of a Multivariable Prediction Model for Individual Prognosis or Diagnosis—Artificial Intelligence
