# Supplementary material for: Convolutional, State-Space and Transformer Liver-Tumor Segmentation Models Trained on Hepatocellular Carcinoma Fail on the Same External Cases: Lesion Size Outweighs Architecture in Cross-Dataset Generalization
Source: Cancers (Basel). 2026 Oct 17;18(20):3362. doi: 10.3390/cancers18203362 (PMC13702596; doi:10.3390/cancers18203362)
Supplement: Supplementary file 1 [file cancers-18-03362-s001.zip › cancers-4595484-supplementary.pdf]

Supplementary Materials

Convolutional, State-Space and Transformer Liver-Tumor Segmentation Models Trained on Hepatocellular Carcinoma Fail on the Same External Cases: Lesion Size Outweighs Architecture in Cross-Dataset Generalization

Hüseyin Kutlu, Cemil Çolak and Sami Akbulut

Contents

Table S1. Completed CLAIM 2024 checklist.

Table S2. Completed TRIPOD+AI checklist.

Table S3. Training configuration and computational environment.

Table S4. Case-level performance for the three architectures.

Table S5. Case-level confidence behavior.

Table S6. Reliability-curve bin statistics.

Figure S1. Flow of cases through the three cohorts.

Table S7. Risk–coverage curves for all architectures, with reference curves.

Table S8. Lesion-wise detection per cohort, architecture and lesion-size stratum.

Table S9. Within-architecture reference and same-fold cross-architecture concordance.

Figure S2. Training dynamics of the transformer arm.

Figure S3. Fold-0 training dynamics, from-scratch versus pretrained encoder.

Tables S4–S9 are presented in landscape orientation at the end of this document. Per-case source data, analysis code and the exact fold assignment are available athttps://github.com/huseyinKutlu/hcc-crossdataset-segmentation and archived at Zenodo (version DOI 10.5281/zenodo.23090049; concept DOI 10.5281/zenodo.23090048, accessed on 1 October 2026).

Table S1. Completed CLAIM 2024 checklist

Item numbering follows the CLAIM 2024 Update [35]. Item descriptions are abbreviated; the official wording is given in the source publication. Items marked NA are accompanied by a justification, as recommended by the 2024 Update.

Section	No.	Item	Status	Reported in
Title / Abstract	1	Identification as a study of AI methodology and category of technology used	Yes	Title; Abstract
Abstract	2	Structured summary of design, methods, results and conclusions	Yes	Abstract
Introduction	3	Scientific and clinical background, including intended use and role of the AI approach	Yes	Section 1
Introduction	4	Study aims, objectives and hypotheses	Yes	Section 1; Section 2.1
Methods — Study design	5	Prospective or retrospective study	Yes	Section 2.1 (retrospective)
Methods — Study design	6	Study goal and type of modelling performed	Yes	Section 2.1
Methods — Data	7	Data sources, with links where available	Yes	Section 2.2; Section 2.8
Methods — Data	8	Inclusion and exclusion criteria	Yes	Section 2.2; Section 3; Figure S1
Methods — Data	9	Data preprocessing	Yes	Section 2.3; Table S3
Methods — Data	10	Selection of data subsets from the raw extracted data	Yes	Section 2.2; Section 2.3
Methods — Data	11	De-identification	NA	All three cohorts are publicly distributed, de-identified archives; no additional de-identification was performed (Section 2.2)
Methods — Data	12	Handling of missing data	Yes	Section 2.6 (undefined distance metrics treated as missing)

Section	No.	Item	Status	Reported in
Methods — Data	13	Image acquisition protocol	Partial	Section 2.2; Table 13 reports slice thickness and in-plane spacing. Full acquisition parameters are not released by the source archives
Methods — Reference standard	14	Definition of the method(s) used to obtain the reference standard	Yes	Section 2.2
Methods — Reference standard	15	Rationale for choosing the reference standard	Yes	Section 2.2; Section 4.6
Methods — Reference standard	16	Source of reference standard annotations, annotator number and qualifications	Partial	Section 2.2; annotations were inherited from the source archives, whose annotator details are given in the cited data descriptors
Methods — Reference standard	17	Annotation of the test set	Yes	Section 2.2 (external annotations used unmodified)
Methods — Reference standard	18	Measures of inter- and intrarater variability	NA	No new annotation was performed; variability of the source annotations was not re-assessed (Section 4.6)
Methods — Data partitions	19	How data were assigned to partitions	Yes	Section 2.3; Table S3
Methods — Data partitions	20	Level at which partitions are disjoint	Yes	Section 2.3 (patient level)
Methods — Testing data	21	Intended sample size and how it was determined	Yes	Section 2.1; Section 4.6
Methods — Model	22	Detailed description of the model	Yes	Section 2.4; Table 1; Table S3; cited architecture references
Methods — Model	23	Software libraries, frameworks and packages with version numbers	Yes	Section 2.8; Table S3
Methods — Model	24	Initialization of model parameters	Yes	Table S3 (random initialization; no transfer learning or pretrained weights)
Methods — Training	25	Details of the training approach	Yes	Section 2.4; Table 1; Table S3
Methods — Training	26	Method of selecting the final model	Yes	Section 2.5; Table S3
Methods — Training	27	Ensembling technique	Yes	Section 2.5; Section 3.10
Methods — Evaluation	28	Metrics of model performance	Yes	Section 2.6
Methods — Evaluation	29	Statistical measures of significance and uncertainty	Yes	Section 2.6; Section 2.8
Methods — Evaluation	30	Robustness or sensitivity analysis	Yes	Sections 3.7, 3.9 and 3.10
Methods — Evaluation	31	Methods for explainability or interpretability	NA	No post hoc explainability method was applied; confidence-based selective prediction is reported instead (Section 2.7)
Methods — Evaluation	32	Evaluation on internal data	Yes	Section 2.3; Section 3.1
Methods — Evaluation	33	Testing on external data	Yes	Section 2.5; Sections 3.2–3.10
Methods — Evaluation	34	Clinical trial registration	NA	Not a clinical trial; retrospective analysis of public imaging archives
Results — Data	35	Numbers of patients or examinations included and excluded	Yes	Section 3; Figure S1
Results — Data	36	Demographic and clinical characteristics of cases in each partition and dataset	Partial	Table 13 reports acquisition and lesion characteristics; patient-level demographics are not distributed

Section	No.	Item	Status	Reported in
				with the source archives (Section 4.6)
Results — Model performance	37	Performance metrics and measures of statistical uncertainty	Yes	Tables 2 and 3; Figure 1; Table S4
Results — Model performance	38	Estimates of performance and their precision	Yes	Table 3; Table 10; Tables S5 and S6
Results — Model performance	39	Failure analysis of incorrect results	Yes	Sections 3.3–3.5; Tables 4–6; Figures 2, 3 and 6; Tables S4 and S5
Discussion	40	Study limitations	Yes	Section 4.6
Discussion	41	Implications for practice, including intended use and clinical role	Yes	Sections 4.2 and 4.3
Other information	42	Reference to the full study protocol or additional technical details	Yes	Section 2.8; this Supplement
Other information	43	Statement on availability of software, trained model and data	Yes	Section 2.8; Data Availability Statement
Other information	44	Sources of funding and other support; role of funders	Yes	Funding; Conflicts of Interest

Table S2. Completed TRIPOD+AI checklist

Item numbering follows the TRIPOD+AI statement [33,34], which comprises 27 items covering the title (1), abstract (2), introduction (3–4), methods (5–17), open science (18), patient and public involvement (19), results (20–24) and discussion (25–27). Item descriptions are abbreviated; the official wording and subitems are given in the source publication.

Section	Item	Reporting recommendation	Reported in
Title	1	Identification of the study as developing or evaluating a prediction model, the target population and the outcome	Title
Abstract	2	Structured summary following the TRIPOD+AI for Abstracts checklist	Abstract
Introduction	3	Background: rationale, existing models, and the intended use and clinical role of the model	Section 1
Introduction	4	Objectives: specific study objectives	Section 1; Section 2.1
Methods	5	Data: source of data, eligibility criteria, study setting and dates	Section 2.2; Figure S1
Methods	6	Participants: key elements of the study setting and the units of analysis	Section 2.2; Section 3
Methods	7	Data preparation: preprocessing, harmonisation and quality control	Section 2.3; Table S3
Methods	8	Outcome: definition of the prediction target and how it was determined	Section 2.2; Section 2.3 (tumor class only)
Methods	9	Predictors: definition and measurement of model inputs	Section 2.3 (resampled, intensity-normalized CT volumes)
Methods	10	Sample size: how the study size was arrived at	Section 2.1; Section 4.6
Methods	11	Missing data: how missing values were handled	Section 2.6
Methods	12	Analytical methods: model type, training and tuning, resampling, and evaluation procedures	Sections 2.3–2.5; Section 2.8; Table S3
Methods	13	Class imbalance: how imbalance in the outcome was addressed	Section 2.4 (compound Dice + cross-entropy loss; nnU-Net foreground oversampling)
Methods	14	Fairness: how fairness across groups was considered, or why it was not assessed	Not assessed — the public archives do not distribute demographic attributes (Section 4.6)
Methods	15	Model output: the form of the model output and how it is interpreted	Section 2.5 (softmax probability maps); Section 2.7
Methods	16	Training, evaluation and comparison: how models were trained, compared and selected	Section 2.4; Section 2.5; Table S3
Methods	17	Model performance: measures of discrimination, calibration and clinical utility	Sections 2.6 and 2.7
Open science	18	Funding, conflicts of interest, protocol, registration, and availability of data and code	Section 2.8; Funding; Conflicts of Interest; Data Availability Statement
PPI	19	Patient and public involvement in the design or conduct of the study	No patient or public involvement; the study reanalyses de-identified public imaging archives
Results	20	Participants: flow of participants, characteristics, and comparison across datasets	Section 3; Table 13; Figure S1
Results	21	Model development: how the final model was arrived at	Sections 2.4, 2.5 and 3.1
Results	22	Model specification: full model or sufficient information to obtain predictions	Section 2.8; Table S3
Results	23	Model performance: performance measures with uncertainty, overall and by subgroup	Tables 2, 3, 5–9; Figures 1–5; Tables S4–S6
Results	24	Model updating: results of any model updating or recalibration	No updating, fine-tuning or recalibration was performed (Section 2.5)
Discussion	25	Interpretation of the results with respect to objectives and other evidence	Sections 4.1, 4.4 and 4.5
Discussion	26	Limitations of the study	Section 4.6

Section	Item	Reporting recommendation	Reported in
Discussion	27	Usability of the model in the context of current care, and implications for future work	Sections 4.2, 4.3 and 5

Table S3. Training configuration and computational environment

Settings are those actually used for each arm and expand Table 1 of the main text. The fourth column describes the pretrained-encoder sensitivity arm (Section 2.4), which differs from the transformer arm only in encoder initialization and was trained for fold 0 only. Two deviations from a fully identical regime were unavoidable and are reported in Section 2.4: U-Mamba required mixed precision to be disabled together with a reduced learning rate and gradient-norm clipping, and SwinUNETR was trained under the reference trainer distributed with the U-Mamba repository, which uses AdamW with cosine annealing and no deep supervision.

Setting	nnU-Net ResEnc-M (CNN)	U-Mamba Bot (SSM)	SwinUNETR (Transformer)	SwinUNETR-pretrained (sensitivity arm, fold 0 only)
Plans / configuration	nnUNetResEncUNetMPlans, 3d_fullres	nnUNetPlans, 3d_fullres	nnUNetPlans, 3d_fullres (feature size 48)	Identical to SwinUNETR
Optimizer	SGD, momentum 0.99, Nesterov	SGD, momentum 0.99, Nesterov	AdamW, weight decay 0.01	AdamW, weight decay 0.01
Learning-rate schedule	poly, initial 0.01	poly, initial 1 × 10−3	cosine, initial 8 × 10−4, minimum 1 × 10−6	cosine, initial 8 × 10−4, minimum 1 × 10−6
Gradient clipping	nnU-Net default	Gradient-norm clipping at 12	Gradient-norm clipping at 12	Gradient-norm clipping at 12
Mixed precision	Enabled	Disabled (non-finite losses in the selective-scan kernels)	Disabled	Disabled
Deep supervision	Yes (5 scales)	Yes (5 scales)	No	No
Epochs per fold	250	250	250	250 (fold 0 only)
Patch size / batch size	64 × 192 × 192 / 2	64 × 192 × 192 / 2	64 × 192 × 192 / 2	64 × 192 × 192 / 2
Target spacing (mm)	1.245 × 0.781 × 0.781	1.245 × 0.781 × 0.781	1.245 × 0.781 × 0.781	1.245 × 0.781 × 0.781
Intensity normalization	nnU-Net CT normalization (0.5th–99.5th foreground percentile clipping, then z-scoring)	Identical	Identical	Identical (differs from the normalization used during pre-training)
Loss	Compound Dice + cross-entropy	Compound Dice + cross-entropy	Compound Dice + cross-entropy	Compound Dice + cross-entropy
Augmentation	nnU-Net defaults	nnU-Net defaults	nnU-Net defaults	nnU-Net defaults
Parameter initialization	Random initialization (framework default); no pretrained weights or transfer learning	Identical	Identical	Swin encoder initialized from the self-supervised weights of Tang et al. [32] (MONAI model_swinvit.pt, 5,050 public CT volumes; 118 of 126 encoder tensors loaded, the remainder being non-trainable buffers); decoder and segmentation head randomly initialized
Cross-validation splits	Shared splits_final.json, patient-disjoint, 83 training / 20 validation per fold	Identical	Identical	Identical (fold 0: 82 training / 21 validation)
Final model	Ensemble of the five fold checkpoints	Identical	Identical	Single fold-0 checkpoint
Inference	nnU-Net sliding window with test-time mirroring; softmax maps retained	Identical	Identical	Sliding window without test-time mirroring; compared only with the fold-0 checkpoints of the other arms under the same setting (Table 16)
Environment	nnU-Net v2.8.1 (Python 3.11, PyTorch 2.4.1 + CUDA 12.1,	U-Mamba fork of nnU-Net	Identical to U-Mamba Bot	Identical to U-Mamba Bot (trainer nnUNetTrainerSwinUNETR_250epochs_pretrained)

Setting	nnU-Net ResEnc-M (CNN)	U-Mamba Bot (SSM)	SwinUNETR (Transformer)	SwinUNETR-pretrained (sensitivity arm, fold 0 only)
	NumPy 2.4.4, SimpleITK 2.5.5)	v2.1.1 (Python 3.10, PyTorch 2.1.2 + CUDA 12.1, NumPy 1.26.4, MONAI 1.3.0, SimpleITK 2.5.5)		
Hardware	Single NVIDIA RTX 6000 Ada (48 GB)	Identical	Identical	Identical
Analysis software	Python 3.13.2 with NumPy 2.5.1, pandas 2.2.3, SciPy 1.16.3, SimpleITK 2.5.3 and statsmodels 0.14.6 (all evaluation and statistical analyses, including the sensitivity analyses)	Identical	Identical	Identical

Figure S1. Flow of cases through the three cohorts

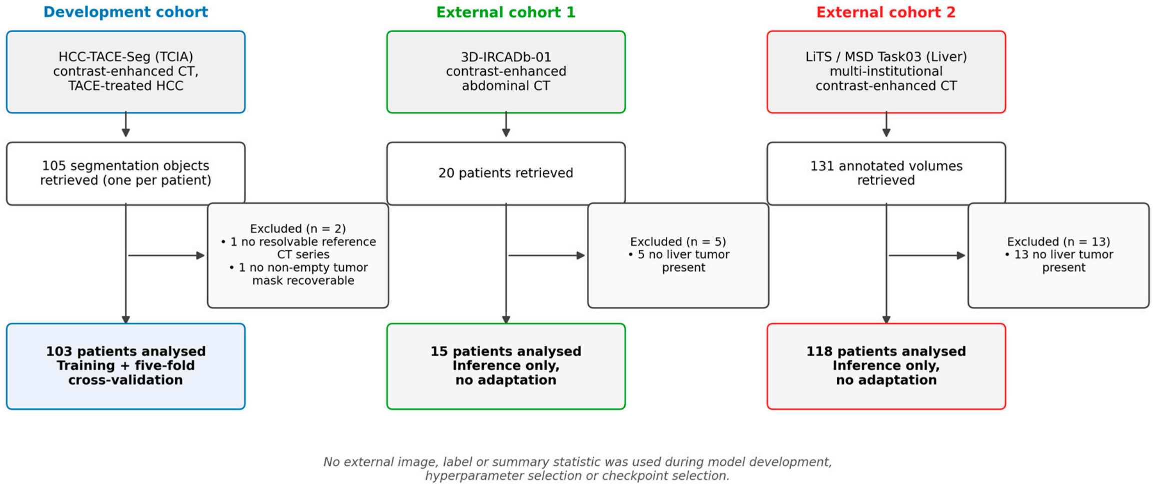

Figure S1. Case selection across the development and the two external cohorts. Cases were excluded only for the reasons shown; none was excluded for image quality. All tumor-bearing cases from both external sources were retained.

Figure S2. Training dynamics of the transformer arm

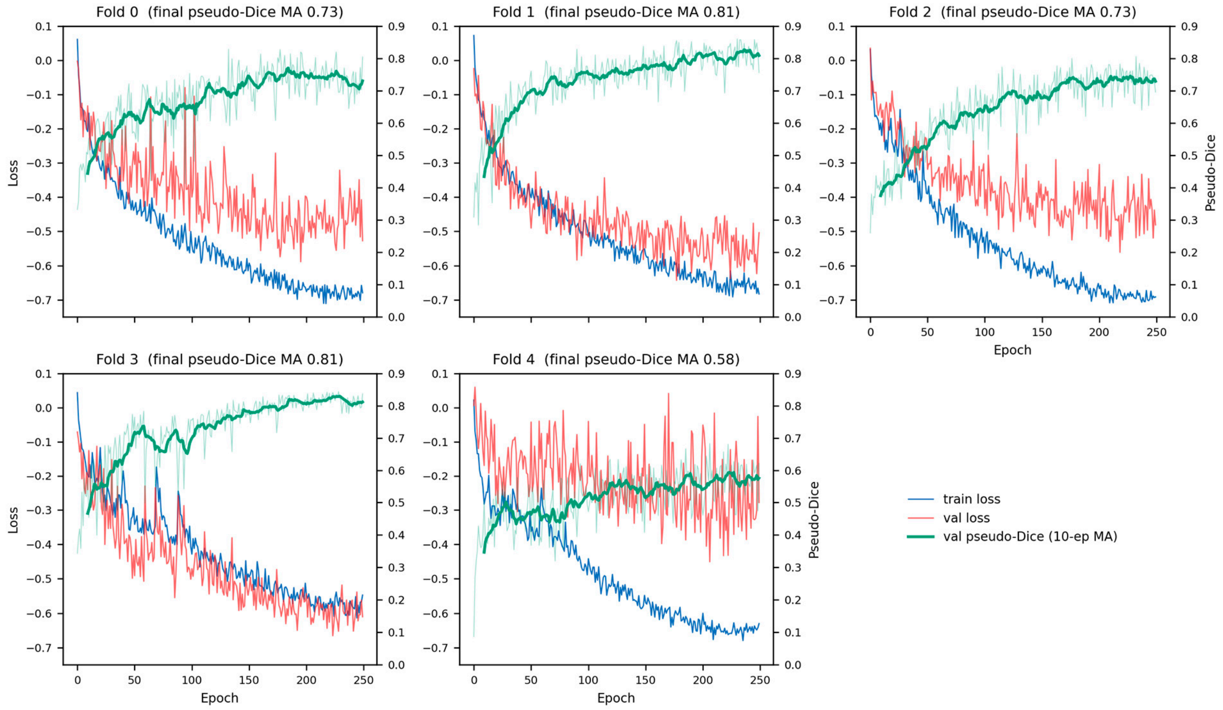

Figure S2. Training dynamics of the transformer arm (SwinUNETR) for each of the five cross-validation folds under the 250-epoch cosine learning-rate schedule (AdamW, $lr_0 = 8\times10^{-4}$). Blue, training loss; red, validation loss; green, validation pseudo-Dice (thin: per epoch; thick: 10-epoch moving average). Validation pseudo-Dice had plateaued by approximately epoch 175–185 in folds 0, 2 and 3, was still rising slowly in fold 1, and remained low and unstable in fold 4, the fold with the lowest internal Dice (Table 2 of the main text). The learning rate reaches zero at epoch 250, so no further gain was possible within the budget.

Figure S3. Fold-0 training dynamics, from-scratch versus pretrained encoder

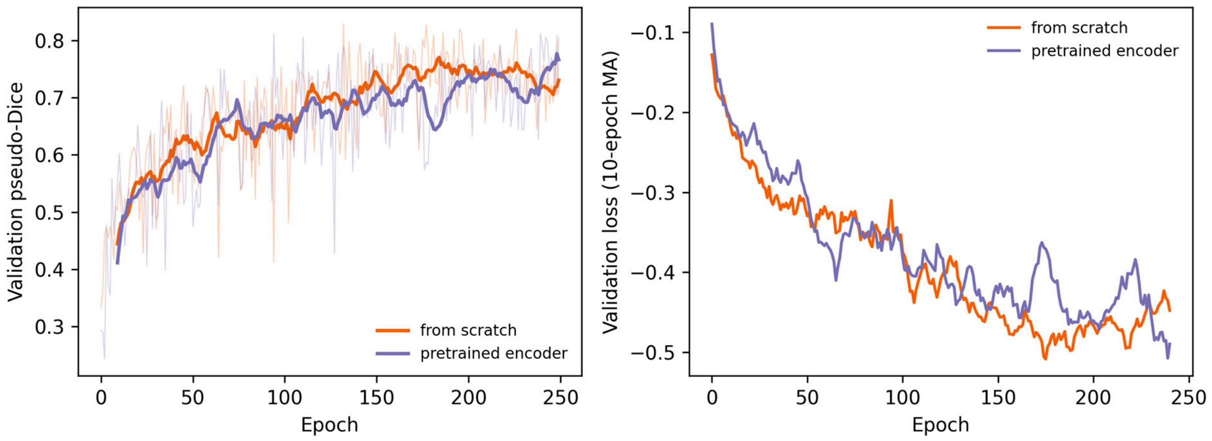

Figure S3. Fold-0 training dynamics of the transformer arm trained from scratch (orange) and with a self-supervised pretrained encoder (purple): validation pseudo-Dice (thin: per epoch; thick: 10-epoch moving average) and validation loss (10-epoch moving average). Pre-training neither accelerated learning nor raised the plateau (Section 3.11 of the main text).

Table S4. Case-level performance for the three architectures

Dice was computed at a probability threshold of 0.5. HD95 and ASSD are symmetric surface distances in millimetres, computed from signed Maurer distance maps using native voxel spacing, and are undefined (—) where either mask was empty. Reference volume is the total tumor volume of the reference annotation. CNN = nnU-Net ResEnc-M; SSM = U-Mamba Bot; Trf. = SwinUNETR.

Case	Cohort	Reference volume (mL)	Dice CNN	Dice SSM	Dice Trf.	HD95 CNN (mm)	HD95 SSM (mm)	HD95 Trf. (mm)	ASSD CNN (mm)	ASSD SSM (mm)	ASSD Trf. (mm)
BIRCAD_001	IRCADb	111.4	0.820	0.845	0.785	56.5	7.6	63.1	8.6	2.5	7.6
BIRCAD_002	IRCADb	13.8	0.000	0.000	0.000	—	—	180.7	—	—	134.8
BIRCAD_003	IRCADb	7.0	0.000	0.000	0.028	—	—	100.3	—	—	50.3
BIRCAD_004	IRCADb	7.4	0.159	0.219	0.063	97.5	81.9	97.5	43.3	33.0	41.0
BIRCAD_005	IRCADb	425.3	0.490	0.587	0.665	81.5	60.1	34.1	22.5	13.9	8.1
BIRCAD_006	IRCADb	10.2	0.000	0.000	0.000	80.8	—	96.2	52.5	—	54.8
BIRCAD_007	IRCADb	60.2	0.856	0.693	0.529	5.5	12.0	112.3	2.8	4.0	26.3
BIRCAD_008	IRCADb	13.0	0.480	0.450	0.361	65.8	106.9	71.8	18.7	37.5	20.7
BIRCAD_009	IRCADb	161.2	0.000	0.003	0.068	—	38.7	141.6	—	29.9	37.2
BIRCAD_010	IRCADb	88.2	0.584	0.666	0.616	44.2	41.1	38.6	11.6	9.6	8.6
BIRCAD_011	IRCADb	1.6	0.000	0.000	0.000	—	—	82.4	—	—	49.8
BIRCAD_012	IRCADb	5.7	0.000	0.000	0.000	59.7	—	139.0	49.6	—	91.1
BIRCAD_013	IRCADb	164.3	0.458	0.590	0.661	96.0	37.5	18.9	31.6	8.9	5.8
BIRCAD_014	IRCADb	6.3	0.000	0.000	0.016	45.9	36.4	132.4	38.3	27.9	71.7
BIRCAD_015	IRCADb	81.9	0.050	0.029	0.034	56.7	75.6	58.6	33.6	43.2	24.6
BLITS_001	LiTS	6.4	0.000	0.000	0.009	—	136.0	53.6	—	83.8	28.9
BLITS_002	LiTS	16.4	0.299	0.315	0.102	38.8	40.0	60.0	9.6	10.9	21.8
BLITS_003	LiTS	14.4	0.660	0.541	0.238	310.9	116.8	69.1	36.7	19.1	31.7
BLITS_004	LiTS	537.2	0.816	0.792	0.696	12.9	15.3	54.4	4.4	4.9	12.3
BLITS_005	LiTS	131.7	0.370	0.350	0.395	27.2	129.8	96.3	8.5	34.8	23.1
BLITS_006	LiTS	17.2	0.862	0.853	0.184	185.4	3.2	208.4	12.8	1.2	85.0
BLITS_007	LiTS	38.8	0.844	0.667	0.526	5.0	121.0	112.1	2.3	28.8	23.2
BLITS_008	LiTS	134.6	0.000	0.000	0.000	—	—	76.0	—	—	35.1
BLITS_009	LiTS	2.9	0.532	0.495	0.393	75.2	77.4	210.0	14.0	16.2	58.3
BLITS_010	LiTS	618.6	0.440	0.408	0.383	24.3	21.5	22.2	7.4	6.5	6.7
BLITS_011	LiTS	33.9	0.005	0.057	0.012	148.0	140.5	55.9	92.1	76.7	24.1
BLITS_012	LiTS	4.8	0.166	0.000	0.012	88.0	—	96.1	40.8	—	48.8
BLITS_013	LiTS	51.6	0.657	0.653	0.550	26.7	69.8	54.7	5.2	13.5	10.4
BLITS_014	LiTS	3.6	0.127	0.244	0.028	273.6	56.9	144.2	82.6	26.1	78.0
BLITS_015	LiTS	0.4	0.000	0.000	0.045	—	—	108.7	—	—	69.5

Case	Cohort	Reference volume (mL)	Dice CNN	Dice SSM	Dice Trf.	HD95 CNN (mm)	HD95 SSM (mm)	HD95 Trf. (mm)	ASSD CNN (mm)	ASSD SSM (mm)	ASSD Trf. (mm)
BLITS_016	LiTS	41.1	0.344	0.587	0.300	48.3	31.1	71.9	18.9	7.5	20.2
BLITS_017	LiTS	266.4	0.044	0.000	0.161	69.0	78.7	104.8	36.9	48.1	38.9
BLITS_018	LiTS	382.5	0.477	0.502	0.359	22.4	34.8	36.8	7.2	8.7	13.2
BLITS_019	LiTS	348.1	0.864	0.847	0.763	17.7	16.9	17.2	4.3	4.8	7.3
BLITS_020	LiTS	0.3	0.000	0.000	0.000	358.2	366.5	171.0	347.3	353.3	109.2
BLITS_021	LiTS	4.3	0.004	0.000	0.000	227.9	228.8	224.3	168.0	171.5	158.7
BLITS_022	LiTS	2.0	0.000	0.000	0.000	—	126.5	374.9	—	66.5	158.9
BLITS_023	LiTS	56.9	0.762	0.736	0.280	38.1	37.2	107.0	5.8	5.8	47.1
BLITS_024	LiTS	191.6	0.666	0.682	0.489	15.2	13.4	115.8	9.2	4.7	30.3
BLITS_025	LiTS	52.9	0.833	0.788	0.599	46.3	47.3	74.3	5.5	6.0	14.8
BLITS_026	LiTS	0.6	0.000	0.000	0.000	—	—	240.3	—	—	139.9
BLITS_027	LiTS	3.4	0.000	0.000	0.000	—	—	155.8	—	—	108.9
BLITS_028	LiTS	0.1	0.000	0.000	0.000	398.5	400.7	425.6	385.6	388.5	184.4
BLITS_029	LiTS	139.5	0.754	0.713	0.264	7.4	47.6	49.6	3.4	8.0	16.5
BLITS_030	LiTS	664.6	0.828	0.741	0.505	16.4	19.6	27.4	4.2	6.0	12.4
BLITS_031	LiTS	17.0	0.272	0.000	0.014	130.2	191.8	129.7	53.3	156.1	44.7
BLITS_032	LiTS	452.6	0.863	0.861	0.799	15.4	15.6	16.1	5.9	4.8	4.8
BLITS_033	LiTS	1.2	0.000	0.645	0.032	246.5	4.2	90.3	230.6	1.3	54.0
BLITS_034	LiTS	0.4	0.000	0.000	0.000	—	279.3	111.8	—	218.6	65.1
BLITS_035	LiTS	147.6	0.719	0.742	0.656	14.6	13.7	55.7	5.6	5.2	8.2
BLITS_036	LiTS	16.7	0.576	0.561	0.250	294.9	296.2	211.4	54.4	62.2	99.4
BLITS_037	LiTS	6.9	0.542	0.630	0.241	45.0	21.3	107.2	13.1	5.8	36.0
BLITS_038	LiTS	8.4	0.289	0.237	0.063	28.1	25.0	122.9	8.5	9.1	38.2
BLITS_039	LiTS	8.5	0.124	0.000	0.000	310.8	—	137.8	81.4	—	81.1
BLITS_040	LiTS	0.9	0.418	0.000	0.002	58.4	—	131.8	21.4	—	78.0
BLITS_041	LiTS	21.4	0.072	0.066	0.082	225.9	220.9	221.6	129.9	128.5	134.7
BLITS_042	LiTS	29.4	0.332	0.332	0.279	33.5	30.8	51.4	14.7	12.4	19.5
BLITS_043	LiTS	22.4	0.197	0.371	0.136	18.2	20.9	106.7	8.4	12.2	48.7
BLITS_044	LiTS	1.0	0.000	0.000	0.000	—	—	183.0	—	—	97.8
BLITS_045	LiTS	0.2	0.000	0.000	0.000	—	—	139.6	—	—	57.5
BLITS_046	LiTS	27.0	0.628	0.679	0.198	8.4	7.4	49.9	3.8	3.5	10.8
BLITS_047	LiTS	71.6	0.636	0.661	0.469	10.1	10.2	61.1	3.6	3.4	13.8
BLITS_048	LiTS	214.3	0.704	0.744	0.717	70.3	67.2	36.1	12.0	10.9	10.7
BLITS_049	LiTS	14.1	0.000	0.000	0.002	—	—	146.7	—	—	66.7
BLITS_050	LiTS	1.3	0.000	0.000	0.000	—	305.7	282.6	—	295.4	176.7

Case	Cohort	Reference volume (mL)	Dice CNN	Dice SSM	Dice Trf.	HD95 CNN (mm)	HD95 SSM (mm)	HD95 Trf. (mm)	ASSD CNN (mm)	ASSD SSM (mm)	ASSD Trf. (mm)
BLITS_051	LiTS	14.3	0.000	0.000	0.017	—	50.2	204.3	—	31.1	72.5
BLITS_052	LiTS	6.7	0.168	0.000	0.064	107.2	—	105.2	44.9	—	35.8
BLITS_053	LiTS	434.7	0.395	0.542	0.613	81.5	76.8	52.7	24.2	18.2	12.7
BLITS_054	LiTS	20.3	0.000	0.000	0.000	116.7	215.2	110.3	78.5	132.0	47.2
BLITS_055	LiTS	39.5	0.551	0.057	0.123	15.6	26.0	104.0	5.5	14.3	33.8
BLITS_056	LiTS	15.0	0.301	0.004	0.000	115.1	166.5	49.6	33.5	93.5	33.6
BLITS_057	LiTS	349.0	0.000	0.007	0.002	163.2	81.0	154.8	133.2	42.2	51.1
BLITS_058	LiTS	987.7	0.796	0.778	0.643	17.0	22.2	34.5	5.3	6.8	12.2
BLITS_059	LiTS	122.5	0.344	0.541	0.479	99.4	51.9	50.7	28.2	14.1	12.7
BLITS_060	LiTS	1.6	0.000	0.000	0.000	—	—	86.5	—	—	63.8
BLITS_061	LiTS	7.3	0.000	0.000	0.000	41.8	47.9	113.6	30.7	37.1	62.4
BLITS_062	LiTS	186.0	0.364	0.462	0.202	128.5	47.2	60.4	42.9	12.5	21.8
BLITS_063	LiTS	4.5	0.000	0.181	0.027	157.0	24.3	152.3	145.0	8.8	77.8
BLITS_064	LiTS	41.4	0.000	0.052	0.009	—	127.0	128.4	—	43.6	55.2
BLITS_065	LiTS	84.6	0.863	0.875	0.803	98.6	99.0	100.8	11.2	10.4	19.0
BLITS_066	LiTS	18.3	0.062	0.120	0.163	83.8	80.6	79.6	46.9	48.1	37.2
BLITS_067	LiTS	0.4	0.000	0.000	0.000	—	49.4	188.6	—	43.3	134.7
BLITS_068	LiTS	6.2	0.119	0.130	0.582	209.3	209.2	22.7	135.1	133.0	5.4
BLITS_069	LiTS	305.1	0.852	0.834	0.657	10.8	10.5	65.6	2.6	2.7	11.9
BLITS_070	LiTS	32.0	0.809	0.799	0.375	53.0	53.2	59.1	7.9	8.2	24.8
BLITS_071	LiTS	6.5	0.154	0.371	0.082	18.0	67.6	205.4	6.6	14.0	127.0
BLITS_072	LiTS	1.8	0.000	0.000	0.000	—	—	154.2	—	—	112.2
BLITS_073	LiTS	3.1	0.000	0.000	0.000	269.3	247.9	288.1	200.8	222.1	185.0
BLITS_074	LiTS	345.8	0.856	0.728	0.696	11.6	143.4	195.0	3.2	18.2	24.5
BLITS_075	LiTS	4.1	0.517	0.141	0.095	145.7	147.3	60.2	28.1	36.8	32.1
BLITS_076	LiTS	1.3	0.000	0.000	0.000	—	235.2	234.2	—	193.6	147.2
BLITS_077	LiTS	0.7	0.000	0.000	0.000	—	—	287.6	—	—	155.7
BLITS_078	LiTS	24.0	0.681	0.701	0.198	312.5	89.8	66.5	36.7	13.7	32.6
BLITS_079	LiTS	14.5	0.716	0.557	0.132	4.7	7.9	206.3	2.6	4.9	122.6
BLITS_080	LiTS	4.2	0.312	0.721	0.257	153.5	47.2	183.0	85.4	5.9	60.5
BLITS_081	LiTS	2.7	0.332	0.000	0.000	49.6	—	160.4	7.3	—	83.4
BLITS_082	LiTS	1.9	0.019	0.130	0.001	82.7	79.3	346.3	59.7	48.2	140.3
BLITS_083	LiTS	216.4	0.925	0.903	0.822	4.3	5.1	99.1	1.4	2.1	15.1
BLITS_084	LiTS	0.5	0.000	0.000	0.000	—	—	78.5	—	—	53.9
BLITS_085	LiTS	5.9	0.489	0.317	0.111	237.2	94.7	72.5	41.8	39.0	47.5

Case	Cohort	Reference volume (mL)	Dice CNN	Dice SSM	Dice Trf.	HD95 CNN (mm)	HD95 SSM (mm)	HD95 Trf. (mm)	ASSD CNN (mm)	ASSD SSM (mm)	ASSD Trf. (mm)
BLITS_086	LiTS	0.7	0.000	0.000	0.000	—	256.3	252.5	—	242.9	139.0
BLITS_087	LiTS	5.4	0.228	0.157	0.025	93.2	100.5	152.3	46.4	59.5	90.1
BLITS_088	LiTS	3.5	0.642	0.603	0.011	77.2	5.7	105.4	12.8	2.3	42.2
BLITS_089	LiTS	18.1	0.704	0.759	0.130	82.7	141.2	67.7	17.8	17.8	34.9
BLITS_090	LiTS	53.9	0.878	0.617	0.453	3.0	53.3	112.4	1.5	13.1	19.5
BLITS_091	LiTS	221.1	0.812	0.789	0.638	13.8	16.9	45.5	3.1	3.8	9.7
BLITS_092	LiTS	25.7	0.861	0.799	0.632	3.1	50.5	65.5	2.4	4.6	9.6
BLITS_093	LiTS	2.1	0.027	0.000	0.024	11.1	—	153.8	6.3	—	73.9
BLITS_094	LiTS	126.8	0.380	0.199	0.000	73.3	210.1	206.7	21.9	74.8	92.9
BLITS_095	LiTS	7.8	0.095	0.714	0.009	132.9	45.2	135.2	89.3	11.3	100.4
BLITS_096	LiTS	266.5	0.782	0.788	0.679	9.6	9.3	11.1	3.6	5.6	3.9
BLITS_097	LiTS	12.9	0.768	0.720	0.052	5.0	5.0	153.6	1.1	1.4	84.9
BLITS_098	LiTS	18.9	0.504	0.394	0.039	70.7	328.1	233.1	12.6	65.3	55.3
BLITS_099	LiTS	17.3	0.538	0.522	0.300	57.7	57.2	109.4	13.4	13.4	27.4
BLITS_100	LiTS	15.0	0.541	0.542	0.107	343.2	82.8	85.7	65.2	33.7	50.4
BLITS_101	LiTS	82.5	0.914	0.914	0.883	2.9	2.9	5.7	0.9	0.9	3.3
BLITS_102	LiTS	3.2	0.731	0.308	0.006	3.0	244.3	154.9	1.3	125.7	85.9
BLITS_103	LiTS	44.3	0.731	0.785	0.681	136.9	5.9	90.2	21.3	2.6	18.4
BLITS_104	LiTS	0.0	0.000	0.000	0.000	304.2	—	328.8	299.8	—	154.7
BLITS_105	LiTS	217.6	0.686	0.709	0.333	12.2	10.8	93.5	4.3	3.9	21.9
BLITS_106	LiTS	4.5	0.000	0.000	0.000	193.2	194.0	157.5	175.2	166.5	90.1
BLITS_107	LiTS	2.0	0.000	0.000	0.000	163.1	—	123.6	144.0	—	72.8
BLITS_108	LiTS	116.2	0.619	0.770	0.571	211.1	13.0	31.5	18.5	5.2	12.7
BLITS_109	LiTS	14.9	0.624	0.445	0.128	325.2	91.5	73.0	44.4	26.4	29.1
BLITS_110	LiTS	122.4	0.016	0.018	0.325	94.8	251.5	63.2	43.3	98.2	17.8
BLITS_111	LiTS	0.9	0.000	0.000	0.001	321.6	329.9	318.4	263.8	313.2	134.7
BLITS_112	LiTS	122.0	0.277	0.157	0.241	56.7	50.6	41.8	18.8	16.9	13.8
BLITS_113	LiTS	30.7	0.531	0.537	0.185	146.3	146.9	91.8	26.7	26.5	40.5
BLITS_114	LiTS	1.1	0.000	0.000	0.000	—	182.5	205.1	—	174.7	140.7
BLITS_115	LiTS	15.8	0.446	0.485	0.073	64.5	66.4	269.2	21.7	21.8	79.3
BLITS_116	LiTS	265.1	0.764	0.749	0.491	9.9	7.6	15.7	3.0	2.6	7.7
BLITS_117	LiTS	229.8	0.761	0.797	0.750	254.9	256.5	314.6	20.8	20.9	33.6
BLITS_118	LiTS	5.2	0.000	0.000	0.000	—	—	65.9	—	—	46.2

Table S5. Case-level confidence behavior

Mean predicted confidence is the mean tumor probability within the predicted tumor region and is undefined (—) where the model produced an empty prediction. Maximum predicted probability is the highest tumor probability anywhere in the volume; read together with the Dice values in Table S4, it identifies cases in which a model was confident while entirely wrong. For selective prediction (Section 2.7 of the main text), cases with an undefined mean confidence were assigned a confidence of zero and are therefore deferred first. Abbreviations as in Table S4.

Case	Cohort	Confidence CNN	Confidence SSM	Confidence Trf.	Maximum probability CNN	Maximum probability SSM	Maximum probability Trf.
BIRCAD_001	IRCADb	0.962	0.962	0.942	1.000	1.000	1.000
BIRCAD_002	IRCADb	—	—	0.809	0.412	0.389	0.996
BIRCAD_003	IRCADb	—	—	0.827	0.026	0.142	0.998
BIRCAD_004	IRCADb	0.736	0.710	0.841	0.990	0.893	0.999
BIRCAD_005	IRCADb	0.899	0.898	0.900	1.000	1.000	1.000
BIRCAD_006	IRCADb	0.728	—	0.720	0.945	0.128	0.956
BIRCAD_007	IRCADb	0.953	0.896	0.844	1.000	0.999	1.000
BIRCAD_008	IRCADb	0.932	0.946	0.822	1.000	1.000	1.000
BIRCAD_009	IRCADb	—	0.701	0.800	0.098	0.922	1.000
BIRCAD_010	IRCADb	0.913	0.926	0.893	1.000	1.000	1.000
BIRCAD_011	IRCADb	—	—	0.755	0.440	0.251	0.991
BIRCAD_012	IRCADb	0.568	—	0.698	0.674	0.174	0.994
BIRCAD_013	IRCADb	0.955	0.931	0.841	1.000	1.000	0.999
BIRCAD_014	IRCADb	0.543	0.518	0.688	0.616	0.593	0.994
BIRCAD_015	IRCADb	0.759	0.620	0.819	0.946	0.770	0.999
BLITS_001	LiTS	—	0.564	0.773	0.422	0.667	0.999
BLITS_002	LiTS	0.824	0.830	0.792	0.998	1.000	1.000
BLITS_003	LiTS	0.917	0.918	0.806	1.000	1.000	1.000
BLITS_004	LiTS	0.959	0.959	0.926	1.000	1.000	1.000
BLITS_005	LiTS	0.848	0.782	0.806	1.000	1.000	1.000
BLITS_006	LiTS	0.933	0.952	0.749	1.000	1.000	0.999
BLITS_007	LiTS	0.955	0.929	0.806	1.000	1.000	1.000
BLITS_008	LiTS	—	—	0.679	0.113	0.328	0.949
BLITS_009	LiTS	0.878	0.861	0.796	0.999	0.999	1.000
BLITS_010	LiTS	0.898	0.858	0.873	1.000	0.999	0.998
BLITS_011	LiTS	0.522	0.627	0.716	0.561	0.844	0.987
BLITS_012	LiTS	0.784	—	0.756	0.996	0.084	0.993
BLITS_013	LiTS	0.899	0.917	0.853	1.000	1.000	1.000
BLITS_014	LiTS	0.726	0.811	0.801	0.999	0.995	0.999

Case	Cohort	Confidence CNN	Confidence SSM	Confidence Trf.	Maximum probability CNN	Maximum probability SSM	Maximum probability Trf.
BLITS_015	LiTS	—	—	0.610	0.047	0.026	0.931
BLITS_016	LiTS	0.821	0.901	0.839	1.000	1.000	1.000
BLITS_017	LiTS	0.653	0.511	0.733	0.880	0.526	0.997
BLITS_018	LiTS	0.868	0.871	0.904	0.999	0.999	1.000
BLITS_019	LiTS	0.958	0.955	0.916	1.000	1.000	1.000
BLITS_020	LiTS	0.689	0.681	0.739	0.943	0.907	0.998
BLITS_021	LiTS	0.864	0.901	0.743	0.998	1.000	0.997
BLITS_022	LiTS	—	0.508	0.796	0.327	0.519	0.999
BLITS_023	LiTS	0.948	0.936	0.806	1.000	1.000	1.000
BLITS_024	LiTS	0.934	0.935	0.883	1.000	1.000	1.000
BLITS_025	LiTS	0.933	0.933	0.909	1.000	1.000	1.000
BLITS_026	LiTS	—	—	0.813	0.084	0.011	0.999
BLITS_027	LiTS	—	—	0.794	0.002	0.007	0.998
BLITS_028	LiTS	0.728	0.777	0.774	0.965	0.971	0.998
BLITS_029	LiTS	0.929	0.935	0.800	1.000	1.000	1.000
BLITS_030	LiTS	0.955	0.921	0.863	1.000	1.000	1.000
BLITS_031	LiTS	0.756	0.523	0.763	0.966	0.571	0.995
BLITS_032	LiTS	0.952	0.936	0.927	1.000	1.000	1.000
BLITS_033	LiTS	0.677	0.678	0.755	0.899	0.888	0.994
BLITS_034	LiTS	—	0.540	0.781	0.115	0.642	0.999
BLITS_035	LiTS	0.954	0.956	0.920	1.000	1.000	1.000
BLITS_036	LiTS	0.908	0.943	0.826	1.000	1.000	1.000
BLITS_037	LiTS	0.946	0.928	0.827	1.000	1.000	1.000
BLITS_038	LiTS	0.931	0.924	0.778	1.000	1.000	0.999
BLITS_039	LiTS	0.604	—	0.744	0.850	0.216	0.991
BLITS_040	LiTS	0.838	—	0.741	0.995	0.007	0.996
BLITS_041	LiTS	0.934	0.910	0.788	1.000	0.998	0.999
BLITS_042	LiTS	0.924	0.933	0.890	0.999	1.000	1.000
BLITS_043	LiTS	0.888	0.881	0.837	0.999	0.998	1.000
BLITS_044	LiTS	—	—	0.801	0.002	0.110	0.999
BLITS_045	LiTS	—	—	0.670	0.003	0.002	0.967
BLITS_046	LiTS	0.877	0.872	0.866	0.999	0.999	1.000
BLITS_047	LiTS	0.921	0.907	0.852	1.000	1.000	1.000
BLITS_048	LiTS	0.913	0.916	0.914	0.999	0.999	1.000
BLITS_049	LiTS	—	—	0.775	0.105	0.077	0.998

Case	Cohort	Confidence CNN	Confidence SSM	Confidence Trf.	Maximum probability CNN	Maximum probability SSM	Maximum probability Trf.
BLITS_050	LiTS	—	0.629	0.708	0.171	0.855	0.998
BLITS_051	LiTS	—	0.799	0.784	0.356	0.982	1.000
BLITS_052	LiTS	0.783	—	0.733	0.979	0.456	0.988
BLITS_053	LiTS	0.894	0.929	0.881	1.000	1.000	1.000
BLITS_054	LiTS	0.746	0.804	0.677	0.990	0.988	0.983
BLITS_055	LiTS	0.871	0.610	0.757	0.998	0.791	0.998
BLITS_056	LiTS	0.734	0.561	0.821	0.952	0.640	0.994
BLITS_057	LiTS	0.703	0.734	0.678	0.927	0.996	0.953
BLITS_058	LiTS	0.918	0.946	0.891	1.000	1.000	1.000
BLITS_059	LiTS	0.855	0.892	0.869	1.000	1.000	1.000
BLITS_060	LiTS	—	—	0.771	0.050	0.007	0.999
BLITS_061	LiTS	0.564	0.609	0.643	0.669	0.777	0.950
BLITS_062	LiTS	0.868	0.818	0.756	0.998	0.999	0.998
BLITS_063	LiTS	0.520	0.678	0.774	0.546	0.881	0.997
BLITS_064	LiTS	—	0.772	0.592	0.495	0.995	0.770
BLITS_065	LiTS	0.965	0.970	0.929	1.000	1.000	1.000
BLITS_066	LiTS	0.864	0.801	0.878	1.000	1.000	1.000
BLITS_067	LiTS	—	0.814	0.784	0.370	0.982	0.996
BLITS_068	LiTS	0.923	0.868	0.800	1.000	0.997	0.995
BLITS_069	LiTS	0.964	0.954	0.903	1.000	1.000	1.000
BLITS_070	LiTS	0.955	0.965	0.796	1.000	1.000	1.000
BLITS_071	LiTS	0.686	0.903	0.767	0.895	0.999	0.999
BLITS_072	LiTS	—	—	0.765	0.027	0.148	0.998
BLITS_073	LiTS	0.776	0.846	0.718	0.991	0.991	0.996
BLITS_074	LiTS	0.947	0.855	0.796	0.999	0.997	0.999
BLITS_075	LiTS	0.867	0.670	0.651	0.998	0.885	0.973
BLITS_076	LiTS	—	0.719	0.868	0.187	0.900	0.999
BLITS_077	LiTS	—	—	0.754	0.032	0.123	0.986
BLITS_078	LiTS	0.911	0.935	0.792	1.000	1.000	1.000
BLITS_079	LiTS	0.948	0.952	0.797	1.000	1.000	1.000
BLITS_080	LiTS	0.795	0.929	0.824	1.000	1.000	1.000
BLITS_081	LiTS	0.768	—	0.679	0.959	0.214	0.968
BLITS_082	LiTS	0.582	0.762	0.840	0.690	0.960	1.000
BLITS_083	LiTS	0.968	0.967	0.896	1.000	1.000	0.999
BLITS_084	LiTS	—	—	0.641	0.103	0.008	0.855

Case	Cohort	Confidence CNN	Confidence SSM	Confidence Trf.	Maximum probability CNN	Maximum probability SSM	Maximum probability Trf.
BLITS_085	LiTS	0.778	0.843	0.798	0.996	1.000	0.999
BLITS_086	LiTS	—	0.740	0.787	0.264	0.927	1.000
BLITS_087	LiTS	0.798	0.775	0.786	1.000	1.000	0.997
BLITS_088	LiTS	0.784	0.877	0.871	0.995	0.999	1.000
BLITS_089	LiTS	0.936	0.951	0.814	1.000	1.000	1.000
BLITS_090	LiTS	0.938	0.890	0.826	1.000	0.999	1.000
BLITS_091	LiTS	0.946	0.928	0.877	1.000	1.000	1.000
BLITS_092	LiTS	0.947	0.917	0.903	1.000	1.000	1.000
BLITS_093	LiTS	0.518	—	0.692	0.544	0.044	0.994
BLITS_094	LiTS	0.842	0.801	0.764	0.996	0.996	0.999
BLITS_095	LiTS	0.778	0.920	0.736	1.000	0.999	0.999
BLITS_096	LiTS	0.922	0.911	0.889	1.000	1.000	1.000
BLITS_097	LiTS	0.924	0.944	0.825	1.000	1.000	1.000
BLITS_098	LiTS	0.884	0.883	0.783	1.000	1.000	0.995
BLITS_099	LiTS	0.882	0.880	0.747	1.000	1.000	0.999
BLITS_100	LiTS	0.905	0.909	0.805	1.000	1.000	1.000
BLITS_101	LiTS	0.964	0.970	0.952	1.000	1.000	1.000
BLITS_102	LiTS	0.953	0.905	0.663	1.000	1.000	0.928
BLITS_103	LiTS	0.933	0.956	0.866	1.000	1.000	1.000
BLITS_104	LiTS	0.539	—	0.675	0.605	0.024	0.987
BLITS_105	LiTS	0.895	0.879	0.808	1.000	1.000	1.000
BLITS_106	LiTS	0.603	0.635	0.737	0.731	0.855	0.995
BLITS_107	LiTS	0.848	—	0.758	0.993	0.018	0.998
BLITS_108	LiTS	0.698	0.876	0.791	0.991	0.995	0.999
BLITS_109	LiTS	0.906	0.929	0.800	1.000	1.000	1.000
BLITS_110	LiTS	0.636	0.886	0.805	0.823	0.997	1.000
BLITS_111	LiTS	0.796	0.860	0.634	0.988	0.991	0.862
BLITS_112	LiTS	0.766	0.738	0.786	1.000	0.998	1.000
BLITS_113	LiTS	0.903	0.899	0.765	1.000	1.000	1.000
BLITS_114	LiTS	—	0.693	0.753	0.200	0.912	0.997
BLITS_115	LiTS	0.930	0.940	0.756	1.000	1.000	1.000
BLITS_116	LiTS	0.924	0.917	0.869	1.000	1.000	1.000
BLITS_117	LiTS	0.939	0.941	0.913	1.000	1.000	1.000
BLITS_118	LiTS	—	—	0.571	0.147	0.046	0.740

Table S6. Reliability-curve bin statistics

Voxels with a predicted tumor probability above 0.01 were assigned to 15 equal-width confidence bins; the trivially confident background below that threshold is excluded. Mean predicted probability and observed tumor frequency are the coordinates plotted in Figure 4. The expected calibration error is the voxel-weighted mean absolute deviation and the maximum calibration error is the largest absolute deviation across populated bins; both are reported in Table 10. A positive deviation indicates under-confidence and a negative deviation over-confidence. Abbreviations as in Table S4.

Cohort	Model	Probability bin	Voxels	Voxels (%)	Mean predicted probability	Observed frequency	Deviation
IRCADb	CNN	0.000–0.067	227,041	25.37	0.027	0.324	+0.297
IRCADb	CNN	0.067–0.133	57,400	6.41	0.096	0.457	+0.361
IRCADb	CNN	0.133–0.200	32,527	3.63	0.165	0.509	+0.345
IRCADb	CNN	0.200–0.267	24,204	2.70	0.232	0.544	+0.313
IRCADb	CNN	0.267–0.333	19,472	2.18	0.299	0.573	+0.273
IRCADb	CNN	0.333–0.400	17,258	1.93	0.366	0.604	+0.237
IRCADb	CNN	0.400–0.467	16,265	1.82	0.433	0.620	+0.187
IRCADb	CNN	0.467–0.533	15,950	1.78	0.500	0.647	+0.147
IRCADb	CNN	0.533–0.600	15,839	1.77	0.567	0.659	+0.092
IRCADb	CNN	0.600–0.667	15,643	1.75	0.634	0.677	+0.043
IRCADb	CNN	0.667–0.733	16,871	1.89	0.700	0.683	-0.018
IRCADb	CNN	0.733–0.800	18,953	2.12	0.768	0.700	-0.068
IRCADb	CNN	0.800–0.867	24,156	2.70	0.835	0.737	-0.098
IRCADb	CNN	0.867–0.933	39,557	4.42	0.903	0.813	-0.090
IRCADb	CNN	0.933–1.000	353,715	39.53	0.990	0.977	-0.013
IRCADb	SSM	0.000–0.067	571,326	41.18	0.026	0.186	+0.160
IRCADb	SSM	0.067–0.133	104,869	7.56	0.094	0.386	+0.292
IRCADb	SSM	0.133–0.200	49,030	3.53	0.164	0.518	+0.354
IRCADb	SSM	0.200–0.267	34,234	2.47	0.232	0.539	+0.307
IRCADb	SSM	0.267–0.333	26,852	1.94	0.299	0.567	+0.268
IRCADb	SSM	0.333–0.400	22,236	1.60	0.366	0.612	+0.246
IRCADb	SSM	0.400–0.467	19,930	1.44	0.433	0.650	+0.217
IRCADb	SSM	0.467–0.533	17,377	1.25	0.499	0.724	+0.224
IRCADb	SSM	0.533–0.600	16,209	1.17	0.567	0.811	+0.245
IRCADb	SSM	0.600–0.667	17,152	1.24	0.634	0.833	+0.199
IRCADb	SSM	0.667–0.733	18,459	1.33	0.700	0.844	+0.144
IRCADb	SSM	0.733–0.800	20,641	1.49	0.768	0.851	+0.083
IRCADb	SSM	0.800–0.867	26,503	1.91	0.835	0.874	+0.039
IRCADb	SSM	0.867–0.933	41,247	2.97	0.904	0.895	-0.009

Cohort	Model	Probability bin	Voxels	Voxels (%)	Mean predicted probability	Observed frequency	Deviation
IRCADb	SSM	0.933–1.000	401,345	28.93	0.989	0.980	-0.010
IRCADb	Trf.	0.000–0.067	3,138,592	37.29	0.028	0.060	+0.031
IRCADb	Trf.	0.067–0.133	981,246	11.66	0.096	0.057	-0.039
IRCADb	Trf.	0.133–0.200	572,446	6.80	0.165	0.059	-0.105
IRCADb	Trf.	0.200–0.267	432,481	5.14	0.232	0.061	-0.171
IRCADb	Trf.	0.267–0.333	357,247	4.24	0.299	0.061	-0.238
IRCADb	Trf.	0.333–0.400	314,883	3.74	0.366	0.060	-0.306
IRCADb	Trf.	0.400–0.467	296,416	3.52	0.433	0.059	-0.374
IRCADb	Trf.	0.467–0.533	293,801	3.49	0.500	0.057	-0.443
IRCADb	Trf.	0.533–0.600	287,718	3.42	0.566	0.059	-0.507
IRCADb	Trf.	0.600–0.667	265,227	3.15	0.633	0.067	-0.566
IRCADb	Trf.	0.667–0.733	235,430	2.80	0.699	0.088	-0.612
IRCADb	Trf.	0.733–0.800	214,309	2.55	0.766	0.117	-0.649
IRCADb	Trf.	0.800–0.867	205,601	2.44	0.833	0.166	-0.668
IRCADb	Trf.	0.867–0.933	219,621	2.61	0.901	0.256	-0.645
IRCADb	Trf.	0.933–1.000	602,117	7.15	0.984	0.740	-0.244
LiTS	CNN	0.000–0.067	11,972,127	26.04	0.028	0.060	+0.032
LiTS	CNN	0.067–0.133	3,131,852	6.81	0.096	0.106	+0.010
LiTS	CNN	0.133–0.200	1,716,868	3.73	0.164	0.135	-0.029
LiTS	CNN	0.200–0.267	1,249,261	2.72	0.232	0.154	-0.078
LiTS	CNN	0.267–0.333	1,034,095	2.25	0.299	0.168	-0.131
LiTS	CNN	0.333–0.400	935,194	2.03	0.366	0.185	-0.181
LiTS	CNN	0.400–0.467	876,972	1.91	0.433	0.202	-0.231
LiTS	CNN	0.467–0.533	859,967	1.87	0.500	0.217	-0.283
LiTS	CNN	0.533–0.600	863,131	1.88	0.567	0.227	-0.339
LiTS	CNN	0.600–0.667	906,882	1.97	0.634	0.232	-0.401
LiTS	CNN	0.667–0.733	977,254	2.13	0.701	0.244	-0.457
LiTS	CNN	0.733–0.800	1,103,989	2.40	0.768	0.260	-0.507
LiTS	CNN	0.800–0.867	1,397,529	3.04	0.835	0.283	-0.552
LiTS	CNN	0.867–0.933	2,140,886	4.66	0.903	0.332	-0.571
LiTS	CNN	0.933–1.000	16,805,085	36.56	0.988	0.677	-0.311
LiTS	SSM	0.000–0.067	12,758,258	26.54	0.028	0.071	+0.043
LiTS	SSM	0.067–0.133	3,592,267	7.47	0.096	0.109	+0.013
LiTS	SSM	0.133–0.200	2,052,079	4.27	0.164	0.131	-0.034
LiTS	SSM	0.200–0.267	1,459,601	3.04	0.232	0.146	-0.085

Cohort	Model	Probability bin	Voxels	Voxels (%)	Mean predicted probability	Observed frequency	Deviation
LiTS	SSM	0.267–0.333	1,180,219	2.46	0.299	0.162	-0.137
LiTS	SSM	0.333–0.400	1,056,445	2.20	0.366	0.179	-0.187
LiTS	SSM	0.400–0.467	975,928	2.03	0.433	0.193	-0.240
LiTS	SSM	0.467–0.533	938,202	1.95	0.500	0.201	-0.299
LiTS	SSM	0.533–0.600	928,631	1.93	0.567	0.212	-0.355
LiTS	SSM	0.600–0.667	977,588	2.03	0.634	0.220	-0.414
LiTS	SSM	0.667–0.733	1,061,557	2.21	0.701	0.234	-0.467
LiTS	SSM	0.733–0.800	1,226,661	2.55	0.768	0.253	-0.515
LiTS	SSM	0.800–0.867	1,564,474	3.25	0.835	0.277	-0.558
LiTS	SSM	0.867–0.933	2,469,628	5.14	0.904	0.329	-0.574
LiTS	SSM	0.933–1.000	15,829,244	32.93	0.986	0.689	-0.297
LiTS	Trf.	0.000–0.067	59,205,809	39.86	0.028	0.018	-0.010
LiTS	Trf.	0.067–0.133	16,214,660	10.92	0.096	0.033	-0.062
LiTS	Trf.	0.133–0.200	8,777,697	5.91	0.164	0.044	-0.120
LiTS	Trf.	0.200–0.267	6,106,342	4.11	0.232	0.052	-0.180
LiTS	Trf.	0.267–0.333	4,764,500	3.21	0.299	0.058	-0.240
LiTS	Trf.	0.333–0.400	4,000,006	2.69	0.366	0.064	-0.301
LiTS	Trf.	0.400–0.467	3,534,637	2.38	0.433	0.071	-0.362
LiTS	Trf.	0.467–0.533	3,267,573	2.20	0.500	0.080	-0.420
LiTS	Trf.	0.533–0.600	3,139,857	2.11	0.567	0.088	-0.478
LiTS	Trf.	0.600–0.667	3,129,717	2.11	0.633	0.099	-0.534
LiTS	Trf.	0.667–0.733	3,270,951	2.20	0.700	0.112	-0.589
LiTS	Trf.	0.733–0.800	3,585,479	2.41	0.767	0.129	-0.639
LiTS	Trf.	0.800–0.867	4,197,013	2.83	0.834	0.150	-0.684
LiTS	Trf.	0.867–0.933	5,683,531	3.83	0.902	0.185	-0.717
LiTS	Trf.	0.933–1.000	19,671,578	13.24	0.981	0.520	-0.462

Table S7. Risk–coverage curves for all architectures, with reference curves

Mean Dice on the retained subset at each coverage level on LiTS (n = 118 at 100%). Confidence ranking: cases ordered by mean predicted confidence, with empty predictions assigned confidence zero and deferred first. Empties removed only: the empty predictions are deferred first and the remaining cases are retained in random order (mean of 1000 permutations). Random deferral: mean of 1000 random orderings. Oracle: cases deferred in ascending order of true Dice. Areas under the risk–coverage curves are given in Table 11 of the main text; the ResEnc-M columns reproduce the reference curves shown there.

Architecture	Coverage	Confidence ranking	Empties removed only	Random deferral	Oracle
ResEnc-M	100%	0.366	0.366	0.366	0.366
	95%	0.386	0.386	0.366	0.386

Architecture	Coverage	Confidence ranking	Empties removed only	Random deferral	Oracle
	90%	0.408	0.408	0.366	0.408
	85%	0.432	0.432	0.366	0.432
	80%	0.459	0.450	0.367	0.460
	75%	0.489	0.450	0.367	0.491
	70%	0.516	0.450	0.367	0.521
	65%	0.542	0.450	0.367	0.559
	60%	0.568	0.450	0.367	0.596
	55%	0.597	0.450	0.367	0.631
	50%	0.627	0.450	0.367	0.664
	45%	0.664	0.450	0.367	0.698
	40%	0.686	0.449	0.367	0.728
	35%	0.708	0.450	0.367	0.756
	30%	0.713	0.451	0.367	0.782
U-Mamba	100%	0.354	0.354	0.354	0.354
	95%	0.373	0.373	0.354	0.373
	90%	0.394	0.394	0.354	0.394
	85%	0.417	0.417	0.354	0.417
	80%	0.444	0.426	0.354	0.444
	75%	0.473	0.426	0.354	0.474
	70%	0.490	0.426	0.354	0.503
	65%	0.524	0.427	0.354	0.542
	60%	0.558	0.426	0.353	0.582
	55%	0.592	0.426	0.353	0.622
	50%	0.619	0.426	0.353	0.658
	45%	0.625	0.426	0.354	0.691
	40%	0.652	0.426	0.354	0.717
	35%	0.687	0.426	0.354	0.742
	30%	0.700	0.426	0.354	0.766
SwinUNETR	100%	0.222	0.222	0.222	0.222
	95%	0.233	0.222	0.222	0.234
	90%	0.245	0.221	0.222	0.247

Architecture	Coverage	Confidence ranking	Empties removed only	Random deferral	Oracle
	85%	0.260	0.221	0.222	0.262
	80%	0.274	0.221	0.222	0.278
	75%	0.287	0.221	0.221	0.297
	70%	0.299	0.221	0.222	0.315
	65%	0.318	0.220	0.222	0.339
	60%	0.344	0.220	0.222	0.366
	55%	0.371	0.220	0.221	0.396
	50%	0.392	0.220	0.221	0.427
	45%	0.404	0.220	0.221	0.462
	40%	0.428	0.220	0.221	0.499
	35%	0.445	0.220	0.220	0.539
	30%	0.495	0.221	0.220	0.583

Table S8. Lesion-wise detection per cohort, architecture and lesion-size stratum

Reference and predicted masks were decomposed into 26-connected components. A reference lesion is counted as detected when the union of overlapping predicted components reaches IoU ≥ 0.1 with it; sensitivity with any voxel overlap is also shown. Lesion-size strata refer to the volume of the individual lesion. False-positive (FP) components are predicted components with no overlap with any reference lesion; FP counts are per architecture and cohort and are therefore repeated across strata. Summarized in Table 9 of the main text.

Cohort	Architecture	Lesion size	n lesions	Sensitivity (IoU ≥ 0.1)	Sensitivity (any overlap)	FP components / case, mean	FP components / case, median
LiTS	ResEnc-M	<1 mL	565	0.032	0.207	0.68	0.0
		1–5 mL	160	0.269	0.469	0.68	0.0
		5–20 mL	110	0.591	0.755	0.68	0.0
		>20 mL	73	0.808	0.918	0.68	0.0
		All	908	0.204	0.377	0.68	0.0
	U-Mamba	<1 mL	565	0.028	0.163	1.02	0.0
		1–5 mL	160	0.244	0.425	1.02	0.0
		5–20 mL	110	0.555	0.718	1.02	0.0
		>20 mL	73	0.795	0.932	1.02	0.0
		All	908	0.192	0.338	1.02	0.0
	SwinUNETR	<1 mL	565	0.021	0.297	37.26	16.5
		1–5 mL	160	0.175	0.531	37.26	16.5
		5–20 mL	110	0.400	0.827	37.26	16.5

Cohort	Architecture	Lesion size	n lesions	Sensitivity (IoU ≥ 0.1)	Sensitivity (any overlap)	FP components / case, mean	FP components / case, median
		>20 mL	73	0.603	0.973	37.26	16.5
		All	908	0.141	0.457	37.26	16.5
3D-IRCADb	ResEnc-M	<1 mL	57	0.000	0.018	0.40	0.0
		1–5 mL	33	0.182	0.242	0.40	0.0
		5–20 mL	17	0.176	0.235	0.40	0.0
		>20 mL	10	0.600	0.600	0.40	0.0
		All	117	0.128	0.162	0.40	0.0
	U-Mamba	<1 mL	57	0.000	0.018	0.40	0.0
		1–5 mL	33	0.091	0.152	0.40	0.0
		5–20 mL	17	0.235	0.353	0.40	0.0
		>20 mL	10	0.600	0.800	0.40	0.0
		All	117	0.111	0.171	0.40	0.0
	SwinUNETR	<1 mL	57	0.035	0.088	60.27	8.0
		1–5 mL	33	0.152	0.242	60.27	8.0
		5–20 mL	17	0.118	0.471	60.27	8.0
		>20 mL	10	0.700	1.000	60.27	8.0
		All	117	0.137	0.265	60.27	8.0

Table S9. Within-architecture reference and same-fold cross-architecture concordance

(a) Within-architecture reference: Spearman ρ of case-level Dice between every pair of the five single-fold checkpoints of the same architecture (single-fold inference, no test-time augmentation); mean, minimum and maximum over the ten pairs. (b) Same-fold cross-architecture concordance: Spearman ρ between the fold-k checkpoints of two architectures, k = 0–4; mean, minimum and maximum over the five folds. Summarized in Table 5 of the main text.

(a) Within-architecture reference

Cohort	Architecture	Folds	Cases	ρ mean	ρ min	ρ max
LiTS	CNN (ResEnc-M)	5	118	0.900	0.857	0.927
LiTS	SSM (U-Mamba)	5	118	0.877	0.815	0.909
LiTS	Transformer (SwinUNETR)	5	118	0.930	0.910	0.949
3D-IRCADb	CNN (ResEnc-M)	5	15	0.890	0.835	0.941
3D-IRCADb	SSM (U-Mamba)	5	15	0.849	0.759	0.922
3D-IRCADb	Transformer (SwinUNETR)	5	15	0.940	0.899	0.971

(b) Same-fold cross-architecture concordance

Cohort	Pair	ϱ mean (same fold)	ϱ min	ϱ max
LiTS	CNN–SSM	0.863	0.786	0.899
LiTS	CNN–Transformer	0.822	0.788	0.844
LiTS	SSM–Transformer	0.849	0.796	0.887
3D-IRCADb	CNN–SSM	0.867	0.782	0.979
3D-IRCADb	CNN–Transformer	0.782	0.667	0.903
3D-IRCADb	SSM–Transformer	0.860	0.760	0.914
